# PIAS3 CircRNA‐Loaded Lipid Nanoparticles Restore ALOX15 SUMOylation to Suppress Cementoblast Ferroptosis and Attenuate Orthodontically Induced Root Resorption

**DOI:** 10.1002/advs.78455

**Published:** 2026-10-26

**Authors:** Yifei Hou, Chongzheng Yan, Xinrui Xu, Zihan Yang, Xinhui Wu, Hongxu Zhao, Xuan Song, Jianing Zhang, Yijing Li, Aohua Yang, Xiamei Lu, Yi Liu

**Affiliations:** ^1^ Department of Orthodontics Cheeloo College of Medicine School and Hospital of Stomatology Shandong University Jinan China; ^2^ Shandong Key Laboratory of Oral Diseases & Shandong Engineering Research Center of Dental Materials and Oral Tissue Regeneration & Shandong Provincial Clinical Research Center For Oral Diseases Jinan China; ^3^ School of Pharmacy Shandong University of Traditional Chinese Medicine Jinan China; ^4^ Shenzhen Research Institute of Shandong University Shenzhen China

**Keywords:** ALOX15, ferroptosis, lipid nanoparticle, OIRR, SUMOylation

## Abstract

Excessive orthodontic compression induces cementoblast dysfunction and contributes to orthodontically induced root resorption (OIRR), yet the molecular mechanisms linking mechanical stress to cementoblast ferroptosis remain unclear. Compression‐induced PIAS3 loss attenuates SUMO3‐dependent ALOX15 SUMOylation and impairs ALOX15 ubiquitination and proteasomal degradation, thereby promoting lipid peroxidation and cementoblast ferroptosis. Based on these findings, PIAS3 circRNA‐loaded uridine‐derived lipid nanoparticles (PIAS3‐OLNPs) were developed, which efficiently restored PIAS3 expression and inhibited cementoblast ferroptosis and subsequent osteoclast differentiation. Moreover, the incorporation of PIAS3‐OLNPs into an injectable F127DA hydrogel prolonged local retention and sustained PIAS3 expression in the gingival sulcus, thereby inhibiting cementoblast ferroptosis and osteoclastogenesis and preserving root integrity in vivo. Collectively, this study establishes PIAS3‐mediated ALOX15 SUMOylation as a key mechanism that restrains cementoblast ferroptosis and demonstrates that restoring this pathway through local PIAS3 circRNA delivery offers a therapeutic strategy for OIRR and potentially other ferroptosis‐related disorders.

## Graphical abstract

Orthodontic compression suppresses PIAS3 expression in cementoblasts, impairing ALOX15 SUMOylation and ubiquitin‐mediated degradation, which leads to ALOX15 accumulation, lipid peroxidation, ferroptosis, and osteoclastogenesis. Local delivery of PIAS3 circRNA‐loaded lipid nanoparticles restores the PIAS3–ALOX15 regulatory pathway, alleviates ferroptotic injury, and preserves root integrity during orthodontic treatment.

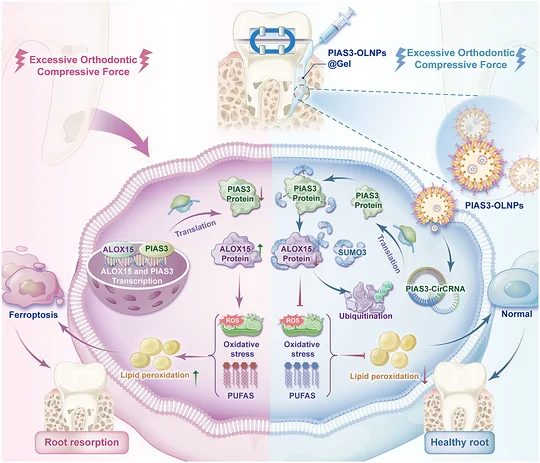

## 1 Introduction

Orthodontically induced root resorption (OIRR) constitutes a common and largely irreversible complication of orthodontic tooth movement that limits safe and predictable treatment outcomes [1, 2]. Severe OIRR can result in substantial root shortening and reduced periodontal support, potentially increasing tooth mobility and compromising long‐term tooth stability [3]. Although controlled mechanical loading is essential for ensuring alveolar bone remodeling, excessive compression can disrupt the periradicular microenvironment and induce pathological resorption of the root surface [4]. Existing clinical management relies primarily on radiographic monitoring, force reduction, or treatment interruption, which may limit further damage but cannot directly reverse cementum and dentin loss or protect cementum‐forming cells from ongoing injury [5, 6]. Importantly, OIRR is not merely a consequence of osteoclast activation but also results from complex interactions among cementoblasts, periodontal ligament cells, immune cells, osteoblasts, and osteoclasts within a mechanically stressed microenvironment [7, 8]. Cementoblasts are particularly important because they maintain cementum integrity and provide a protective barrier against root resorption. Under excessive compression, however, these cells may lose this protective phenotype, thereby promoting matrix degradation, tissue injury, and osteoclastogenic signaling [9]. Therefore, defining the molecular mechanisms that link orthodontic force to cementoblast dysfunction is essential for developing effective root‐protective therapies.

Ferroptosis represents an iron‐dependent form of regulated cell death driven by phospholipid peroxidation and may provide a mechanistic link among mechanical stress, oxidative injury, and tissue remodeling in OIRR [10]. Emerging evidence indicates that compressive and inflammatory stimuli induce iron accumulation, reactive oxygen species (ROS) production, lipid peroxidation, mitochondrial damage, and glutathione peroxidase 4 (GPX4) suppression in periodontal cells, whereas pharmacological inhibition of ferroptosis attenuates experimental root resorption [11, 12]. Arachidonate 15‐lipoxygenase (ALOX15), a lipid‐oxidizing enzyme that catalyzes polyunsaturated fatty acid (PUFA) peroxidation, may represent a key mediator of force‐induced cementoblast ferroptosis [13]. Notably, the abundance and stability of ALOX15 may be regulated not only transcriptionally but also through post‐translational modifications, including ubiquitination and SUMOylation [14, 15]. In eukaryotic cells, intracellular protein homeostasis is governed primarily by two evolutionarily conserved degradative systems, i.e., the ubiquitin–proteasome system and the autophagy–lysosome pathway [16]. SUMOylation can modulate protein stability and degradation through crosstalk with these two proteolytic systems. Because SUMOylation is reversible, substrate SUMOylation reflects a dynamic balance between SUMO conjugation and deSUMOylation. Although SUMOylation of ferroptosis‐related proteins has been investigated in other diseases [17, 18], whether SUMOylation regulates ALOX15‐driven ferroptosis during OIRR remains unknown. Protein inhibitor of activated STAT 3 (PIAS3) constitutes a substrate‐selective SUMO E3 ligase that directs SUMO conjugation to defined protein targets, thereby reshaping their interactions, activity, and turnover. Proteome‐scale mapping has revealed notable substrate preferences among SUMO E3 ligases, including PIAS3, emphasizing their capacity for pathway‐specific regulation [19]. Recent studies have also indicated that PIAS3‐dependent SUMOylation can facilitate the coupling of stress or metabolic signaling to ubiquitin‐associated substrate destabilization and osteogenic differentiation [20, 21]. This regulatory profile renders PIAS3 a plausible upstream determinant of ferroptosis sensitivity through the control of the abundance of lipid‐peroxidizing enzymes. However, whether orthodontic compression disrupts PIAS3‐dependent regulation of ALOX15 in cementoblasts remains unknown.

Moreover, OIRR occurs mostly on the pressure side of the root, making it difficult for systemically applied antioxidants or ferroptosis inhibitors to reach the area around the tooth root. Local delivery of RNA nanomedicines has increasingly emerged as a promising therapeutic strategy to enhance therapeutic protein expression in diseased tissues while reducing systemic adverse effects [22]. Circular RNA (circRNA) is attractive for this purpose because its covalently closed structure increases resistance to exonuclease‐mediated degradation and supports durable protein expression [23]. Lipid nanoparticles (LNPs) can protect RNA and facilitate intracellular delivery, whereas injectable hydrogels can serve as local depots that prolong retention and provide sustained release in the anatomically confined gingival sulcus.

Here, we demonstrate that ALOX15 accumulation during OIRR is associated with both *Alox15* transcriptional upregulation and impaired PIAS3‐mediated protein degradation in cementoblasts (Scheme 1). Compressive stress suppresses PIAS3 expression, thereby reducing SUMO3 conjugation to ALOX15 and its subsequent ubiquitination and proteasomal degradation. The resulting stabilization of ALOX15 amplifies lipid peroxidation, iron accumulation, and ferroptotic injury while promoting a paracrine osteoclastogenic response that aggravates root resorption. Restoring PIAS3 expression in cementoblasts with OA‐C12‐based LNPs (OLNPs) that carry PIAS3‐encoding circRNA (PIAS3 circRNA) re‐establishes ALOX15 SUMOylation and turnover, restores antioxidant defenses, and preserves mitochondrial and redox homeostasis, thereby suppressing cementoblast ferroptosis and osteoclast differentiation. Furthermore, the incorporation of PIAS3‐OLNPs into an injectable Pluronic F127 diacrylate (F127DA) hydrogel prolongs their local retention on the compression side of the root and notably attenuates ferroptosis and root resorption in vivo. Collectively, these findings identify impaired PIAS3‐dependent ALOX15 degradation as an important post‐translational mechanism that amplifies compression‐induced ALOX15 accumulation and establish localized PIAS3 circRNA delivery as a mechanism‐based strategy for protecting root integrity during orthodontic treatment.

**SCHEME 1:**
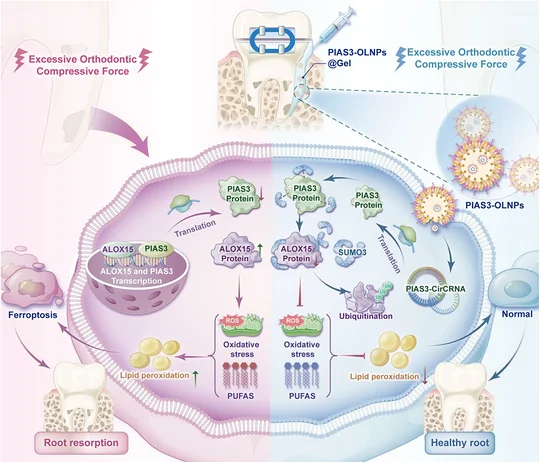
Schematic illustration of the PIAS3–ALOX15 axis in orthodontic compression‐induced ferroptosis and its therapeutic restoration. Excessive orthodontic compression is associated with increased *Alox15* transcription and reduced PIAS3 expression. PIAS3 loss decreases ALOX15 SUMOylation, ubiquitination, and proteasomal degradation. These transcriptional and post‐translational alterations jointly promote ALOX15 accumulation, lipid peroxidation, cementoblast ferroptosis, and root resorption. Local delivery of PIAS3 circRNA via PIAS3‐OLNPs@Gel restores PIAS3 expression and ALOX15 degradation, thereby suppressing ferroptosis and preserving root integrity.

## 2 Results

### 2.1 Ferroptosis Inhibition Attenuates OIRR

To determine whether ferroptosis contributes to OIRR and involves cementoblasts, we established a rat orthodontic tooth movement model with local ferrostatin‐1 (Fer‐1) administration (Figure 1A,B). Micro‐computed tomography (micro‐CT) showed pronounced root resorption in the OIRR group, which was significantly attenuated by Fer‐1 (Figure 1C and Figure S1B). Tooth movement distance was comparable between the OIRR and OIRR + Fer‐1 groups, indicating that the protective effect of Fer‐1 was not attributable to reduced orthodontic tooth displacement (Figure S1A,C).

**FIGURE 1:**
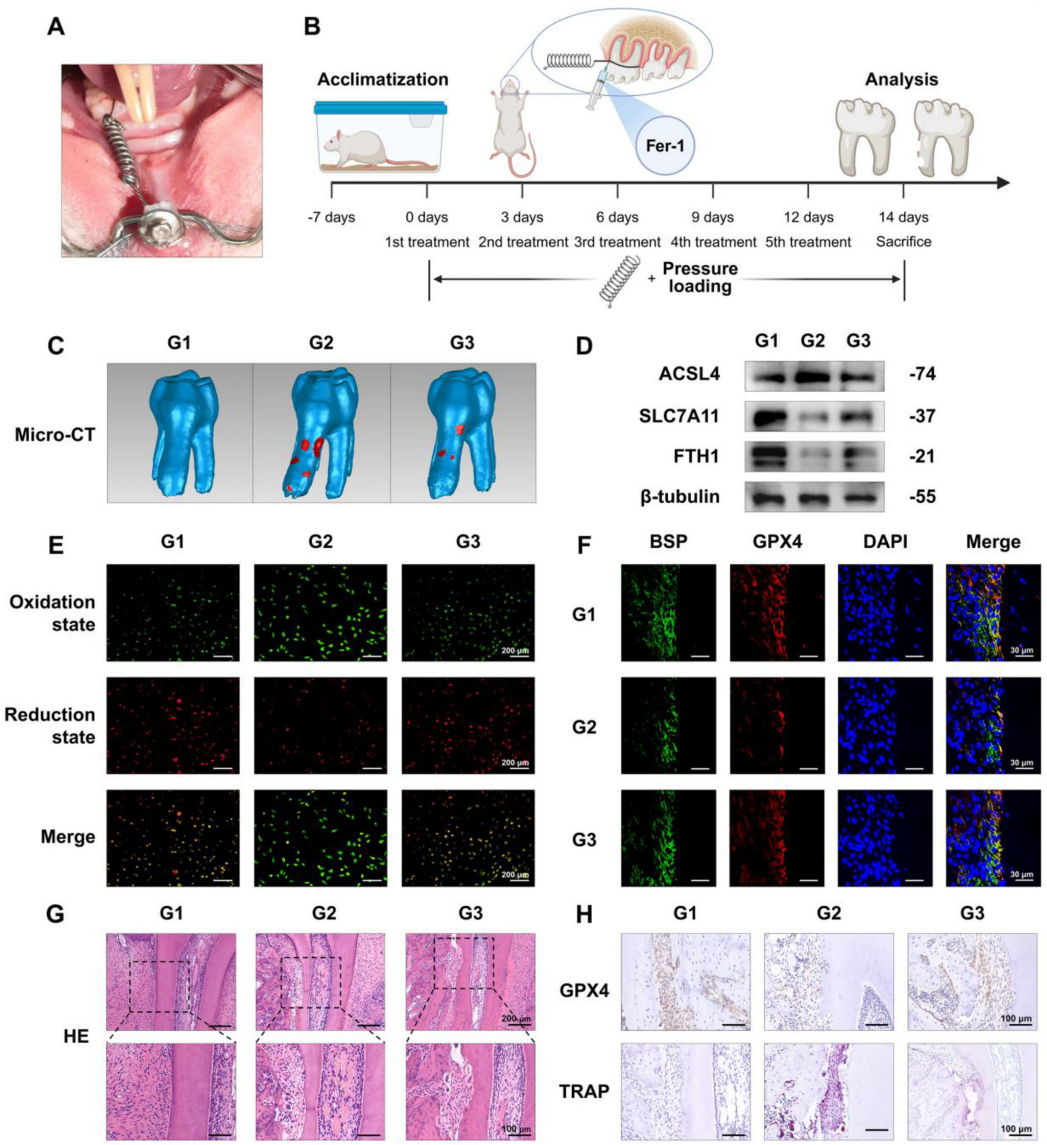
Ferroptosis inhibition attenuates OIRR in vivo. (A) Representative intraoral photograph of the rat orthodontic force‐loading appliance. (B) Schematic of the experimental timeline. (C) Representative micro‐CT reconstructions of maxillary first molar roots. Red pseudocolored areas indicate root resorption lacunae. (D) Western blot analysis of ACSL4, SLC7A11, and FTH1 in cementoblast‐enriched primary cells. (E) BODIPY 581/591 C11 staining showing lipid oxidation and reduction states in cementoblast‐enriched primary cells. Scale bars: 200 µm. (F) Immunofluorescence staining of BSP, GPX4, and DAPI in periodontal tissues. Scale bars: 30 µm. (G) H&E staining of root and periodontal tissues, with magnified views of the boxed regions. Scale bars: 200 µm in the upper panels and 100 µm in the lower magnified panels. (H) Immunohistochemical staining of GPX4 and TRAP staining in periodontal tissues. Scale bars: 100 µm. G1, Control; G2, OIRR; G3, OIRR + Fer‐1.

Cementoblast‐enriched primary cell populations were obtained from Control, OIRR, and OIRR + Fer‐1 rats using the isolation and culture workflow shown in Figure S2. Bone sialoprotein (BSP) positivity and cluster of differentiation 146 (CD146) negativity supported the enrichment of cementoblasts (Figure S1D). Compared with those from Control rats, cementoblast‐enriched primary cells isolated from OIRR rats exhibited increased acyl‐CoA synthetase long‐chain family member 4 (ACSL4) and decreased solute carrier family 7 member 11 (SLC7A11) and ferritin heavy chain 1 (FTH1) expression. Lipid oxidation, malondialdehyde (MDA), and Fe^2^^+^ levels were increased, whereas the GSH/GSSG ratio was reduced; these changes were substantially reversed by Fer‐1 (Figure 1D,E and Figure S1E–G). Dual immunofluorescence further revealed reduced GPX4 in BSP‐positive cells in periodontal tissues during OIRR, with partial restoration after Fer‐1 treatment (Figure 1F). Consistently, Fer‐1 alleviated root surface defects, restored GPX4 expression, and reduced TRAP‐positive cells in vivo (Figure 1G,H).

In OCCM‐30 cells, GPX4 expression decreased markedly by 24 h of compression and showed little further reduction thereafter. Thus, 24 h was selected for subsequent in vitro experiments (Figure S3A). Representative bright‐field images show cell shrinkage after compression (Figure S3B). Under this condition, compression decreased SLC7A11 and FTH1, increased ACSL4, and induced lipid peroxidation, MDA accumulation, Fe^2^^+^ overload, and GSH/GSSG depletion. These effects were partially reversed by Fer‐1 (Figure S3C–H), further supporting compression‐induced ferroptosis in cementoblasts.

### 2.2 RNA Sequencing (RNA‐seq) Reveals Ferroptosis and PIAS3 Dysregulation in OIRR

RNA‐seq was performed on compression‐side periodontal tissues from control and OIRR rats to identify molecular alterations associated with ferroptosis. Kyoto Encyclopedia of Genes and Genomes (KEGG) analysis showed enrichment of arachidonic acid metabolism, ferroptosis, glutathione metabolism, inflammatory signaling, and osteoclastogenesis‐related pathways (Figure 2A). Gene Ontology (GO) analysis similarly highlighted lipid metabolism, glutathione metabolism, lipid droplets, peroxisomes, and iron ion binding (Figure S4).

**FIGURE 2:**
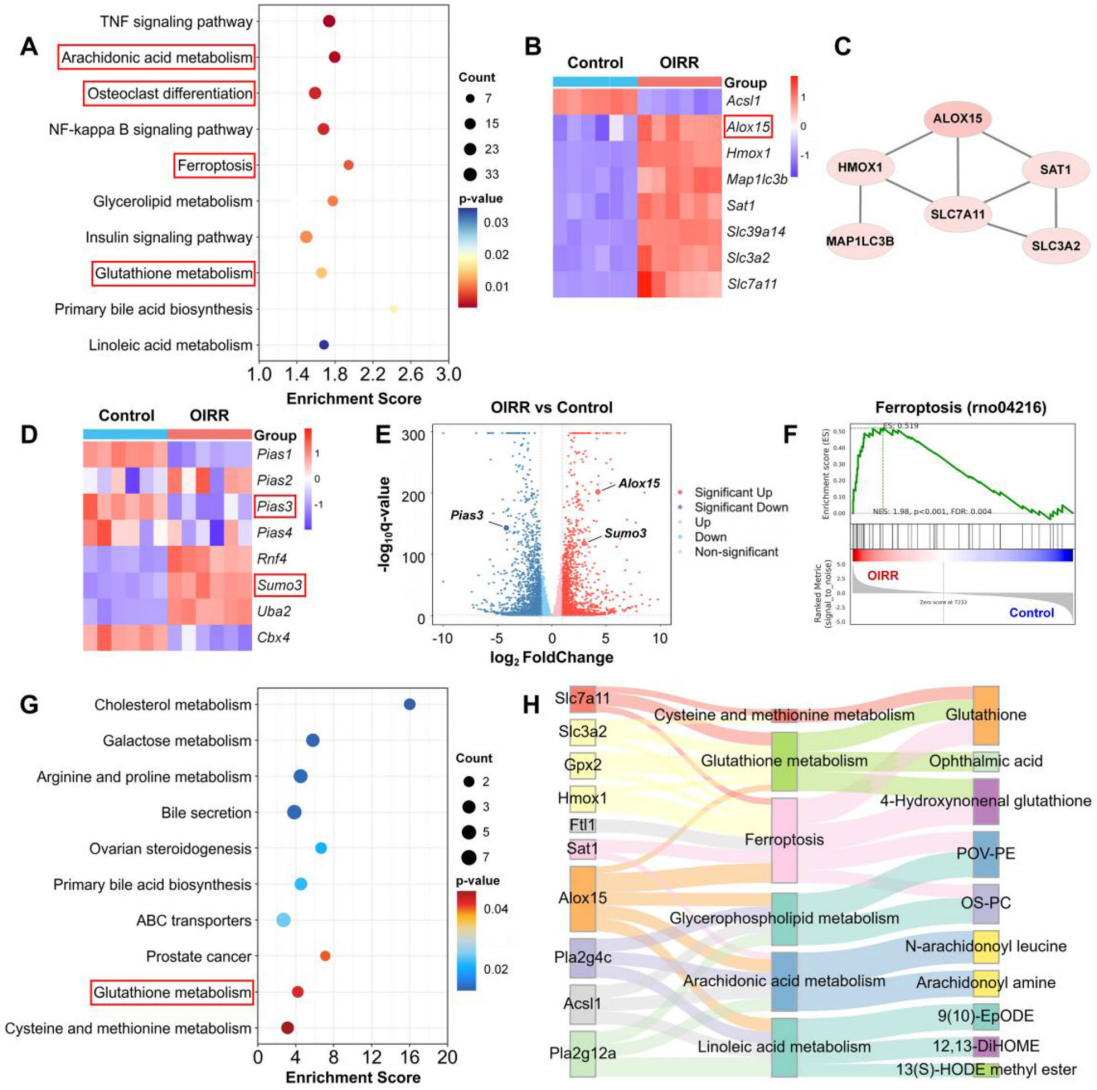
Integrated transcriptomic and metabolomic analysis. (A) KEGG enrichment of DEGs. (B) Heatmap of ferroptosis‐related genes. (C) ALOX15‐centered protein‐interaction network. (D) Heatmap of PIAS‐family and SUMOylation‐related genes. (E) Volcano plot of DEGs. (F) GSEA of ferroptosis. (G) KEGG enrichment of differential metabolites. (H) Sankey diagram linking ferroptosis‐related genes, metabolic pathways, and metabolites.

Among ferroptosis‐associated genes, *Alox15* was markedly upregulated in OIRR tissues, together with heme oxygenase 1 (*Hmox1*), microtubule‐associated protein 1 light chain 3 beta (*Map1lc3b*), spermidine/spermine N1‐acetyltransferase 1 (*Sat1*), solute carrier family 39 member 14 (*Slc39a14*), solute carrier family 3 member 2 (*Slc3a2*), and *Slc7a11*, whereas acyl‐CoA synthetase long‐chain family member 1 (*Acsl1*) was reduced (Figure 2B). STRING database analysis placed ALOX15 within an interaction network containing HMOX1, SAT1, SLC7A11, SLC3A2, and MAP1LC3B (Figure 2C). Analysis of SUMOylation‐related genes showed that *Pias3* decreased by approximately 2.33‐fold, while protein inhibitor of activated STAT 2 (*Pias2*) increased modestly and protein inhibitor of activated STAT 4 (*Pias4*) remained largely unchanged. In contrast, small ubiquitin‐like modifier 3 (*Sumo3*), ubiquitin‐like modifier activating enzyme 2 (*Uba2*), and ring finger protein 4 (*Rnf4*) were increased, whereas chromobox 4 (*Cbx4*) was decreased (Figure 2D), arguing against global suppression of the SUMOylation machinery. Overall, 1538 genes were upregulated, and 1873 were downregulated, and gene set enrichment analysis (GSEA) confirmed positive enrichment of the ferroptosis pathway in OIRR tissues (Figure 2E,F).

Because bulk periodontal RNA‐seq could not define the cellular origin of these transcriptional changes, key targets were further examined in cementoblast‐enriched primary cells isolated from rats. OIRR‐derived cementoblasts exhibited increased *Alox15* and decreased *Pias3* and *Slc7a11* mRNA expression, as determined by reverse transcription quantitative polymerase chain reaction (RT‐qPCR), with consistent changes at the protein level detected by Western blotting (Figure S5A–D). ALOX15 protein was also markedly increased after 24 h of compression in OCCM‐30 cells and remained elevated at 48 h (Figure S6). These findings prompted further investigation of the PIAS3–ALOX15 regulatory axis.

### 2.3 Untargeted Metabolomics Reveals Ferroptosis‐Related Metabolic Remodeling in OIRR

Untargeted metabolomics of compression‐side periodontal tissues revealed extensive metabolic remodeling in OIRR, with 232 metabolites increased and 392 decreased (Figure S7A). Metabolite set enrichment analysis (MSEA) identified arachidonic acid and glutathione metabolism among the significantly altered pathways (Figure S7B). Hierarchical clustering further demonstrated accumulation of arachidonic acid‐derived oxylipins and oxidized phospholipids together with depletion of antioxidant metabolites, including glutathione and ophthalmic acid (Figure S7C). KEGG analysis similarly showed enrichment of glutathione, cysteine, and methionine metabolism pathways (Figure 2G).

Integrated transcriptomic–metabolomic analysis revealed coordinated changes in ferroptosis‐associated genes, metabolic pathways, and downstream metabolites (Figure 2H). ALOX15 upregulation was associated with enhanced arachidonic acid metabolism and accumulation of lipid peroxidation products, whereas alteration in SLC7A11 was linked to disrupted glutathione metabolism. The ALOX15‐centered correlation network was also associated with arachidonic acid derivatives, bioactive phospholipids, and bile acid species (Figure S7D). These findings indicate that OIRR is characterized by impaired antioxidant capacity and a lipid peroxidation‐prone metabolic state.

### 2.4 PIAS3 Interacts with and Destabilizes ALOX15

Given the opposite expression patterns of PIAS3 and ALOX15, we examined whether PIAS3 regulates ALOX15 protein stability. Reciprocal co‐immunoprecipitation (co‐IP) in OCCM‐30 cells co‐expressing HA‐PIAS3 and Flag‐ALOX15 confirmed an interaction between the two proteins (Figure 3A and Figure S8). Cycloheximide (CHX) chase assays showed that PIAS3 overexpression accelerated ALOX15 degradation and shortened its half‐life from approximately 8 to 4 h (Figure S9A,B). After confirming *PIAS3* knockdown efficiency by RT‐qPCR (Figure S9C), we found that PIAS3 depletion delayed ALOX15 degradation and prolonged its half‐life (Figure S9D,E).

**FIGURE 3:**
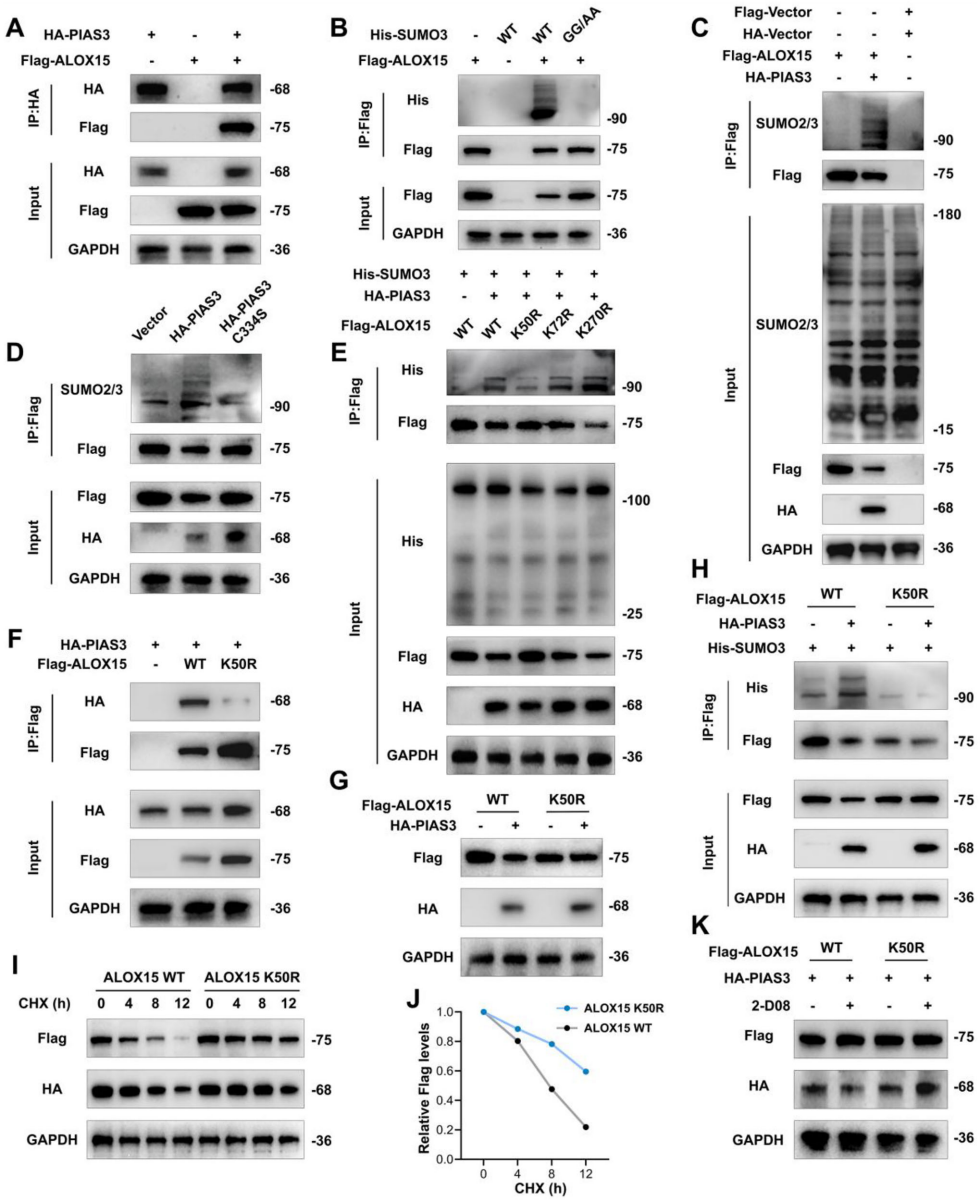
PIAS3 promotes SUMOylation of ALOX15. (A) Co‐IP of HA‐PIAS3 and Flag‐ALOX15. (B) ALOX15 SUMOylation with wild‐type or conjugation‐defective SUMO3 (GG/AA). (C) PIAS3‐mediated SUMOylation of ALOX15. (D) Effect of wild‐type and C334S mutant PIAS3 on SUMOylation of ALOX15. (E) SUMOylation of wild‐type ALOX15 and K50R, K72R, or K270R mutants. (F) PIAS3 binding to wild‐type or K50R ALOX15. (G) Effect of PIAS3 on ALOX15 abundance in wild‐type and K50R mutant. (H) Effect of PIAS3 on SUMO3 modification of wild‐type and K50R mutant ALOX15. (I, J) CHX chase of wild‐type and K50R ALOX15. (K) Effect of the SUMOylation inhibitor 2‐D08 on PIAS3‐mediated ALOX15 degradation.

Because *Alox15* mRNA was increased in OIRR tissues and cementoblast‐enriched primary cells (Figure 2B and Figure S5A), we further assessed whether PIAS3 deficiency directly affects *Alox15* transcription. PIAS3 knockdown did not significantly alter *Alox15* mRNA but further increased compression‐induced ALOX15 protein accumulation (Figure S10A,B). A similar increase was observed for exogenous Flag‐ALOX15 under compression (Figure S10C). Together with the PIAS3‐dependent changes in ALOX15 half‐life observed in CHX chase assays (Figure S9A,B,D,E), these findings indicate that transcriptional upregulation and impaired PIAS3‐mediated degradation jointly contribute to ALOX15 accumulation.

Protein–protein docking predicted a potential PIAS3–ALOX15 interaction interface involving several noncovalent contacts. Predicted interacting residues included Arg63, Ser152, Ser545, and Glu538 of PIAS3 and His30, Gln125, Lys50, and Lys156 of ALOX15, respectively (Figure S11), providing a structural basis for subsequent analysis of ALOX15 post‐translational modification.

### 2.5 PIAS3 Promotes SUMO3‐Mediated SUMOylation of ALOX15

To determine whether ALOX15 undergoes SUMOylation, Flag‐ALOX15 was co‐expressed with wild‐type His‐SUMO3 or a conjugation‐defective SUMO3 GG/AA mutant. Wild‐type SUMO3 produced a characteristic high‐molecular‐weight smear of ALOX15, whereas this signal was markedly reduced with the mutant construct (Figure 3B). PIAS3 overexpression further enhanced SUMO2/3 modification of ALOX15, whereas the catalytically inactive PIAS3 C334S mutant largely lost this effect (Figure 3C,D), supporting that PIAS3 may act as an E3 SUMO ligase for ALOX15.

Because substrate SUMOylation is dynamically balanced by deSUMOylation, representative SUMO‐specific proteases (SENPs) were examined after compression. SENP1, SENP2, SENP3, and SENP5 protein levels remained unchanged (Figure S12), suggesting that the compression‐associated reduction in ALOX15 SUMOylation was unlikely to result primarily from increased deSUMOylase abundance.

Four prediction websites identified Lys50, Lys72, and Lys270 as candidate ALOX15 SUMOylation sites (Figure S13A). Mutation of Lys50 to arginine markedly reduced PIAS3‐mediated SUMO3 modification, whereas K72R and K270R showed SUMOylation levels comparable to wild‐type ALOX15 (Figure 3E). Lys50 was highly conserved across 10 mammalian species (Figure S13B), and the K50R mutation also weakened the interaction between PIAS3 and ALOX15 (Figure 3F).

PIAS3 overexpression reduced wild‐type ALOX15 abundance but had little effect on K50R ALOX15 (Figure 3G). Consistently, robust SUMO3 modification was detected on wild‐type ALOX15 in the presence of PIAS3 but was barely detectable on K50R (Figure 3H). CHX chase analysis further showed that the K50R mutant had a prolonged half‐life and slower degradation than wild‐type ALOX15 (Figure 3I,J). In addition, the SUMOylation inhibitor 2‐D08 restored PIAS3‐suppressed wild‐type ALOX15 levels but had minimal effect on K50R (Figure 3K). These results support Lys50 as a major PIAS3‐dependent SUMO3 acceptor site involved in ALOX15 degradation.

### 2.6 PIAS3 Promotes Ubiquitination and Proteasomal Degradation of ALOX15

We next investigated the degradation pathway downstream of PIAS3‐mediated ALOX15 SUMOylation. PIAS3 overexpression reduced endogenous ALOX15, and this effect was partially reversed by the proteasome inhibitor MG132 in a dose‐dependent manner (Figure 4A,B). In contrast, chloroquine did not restore ALOX15 abundance (Figure 4C,D), indicating that proteasomal rather than lysosomal degradation predominated under these conditions.

**FIGURE 4:**
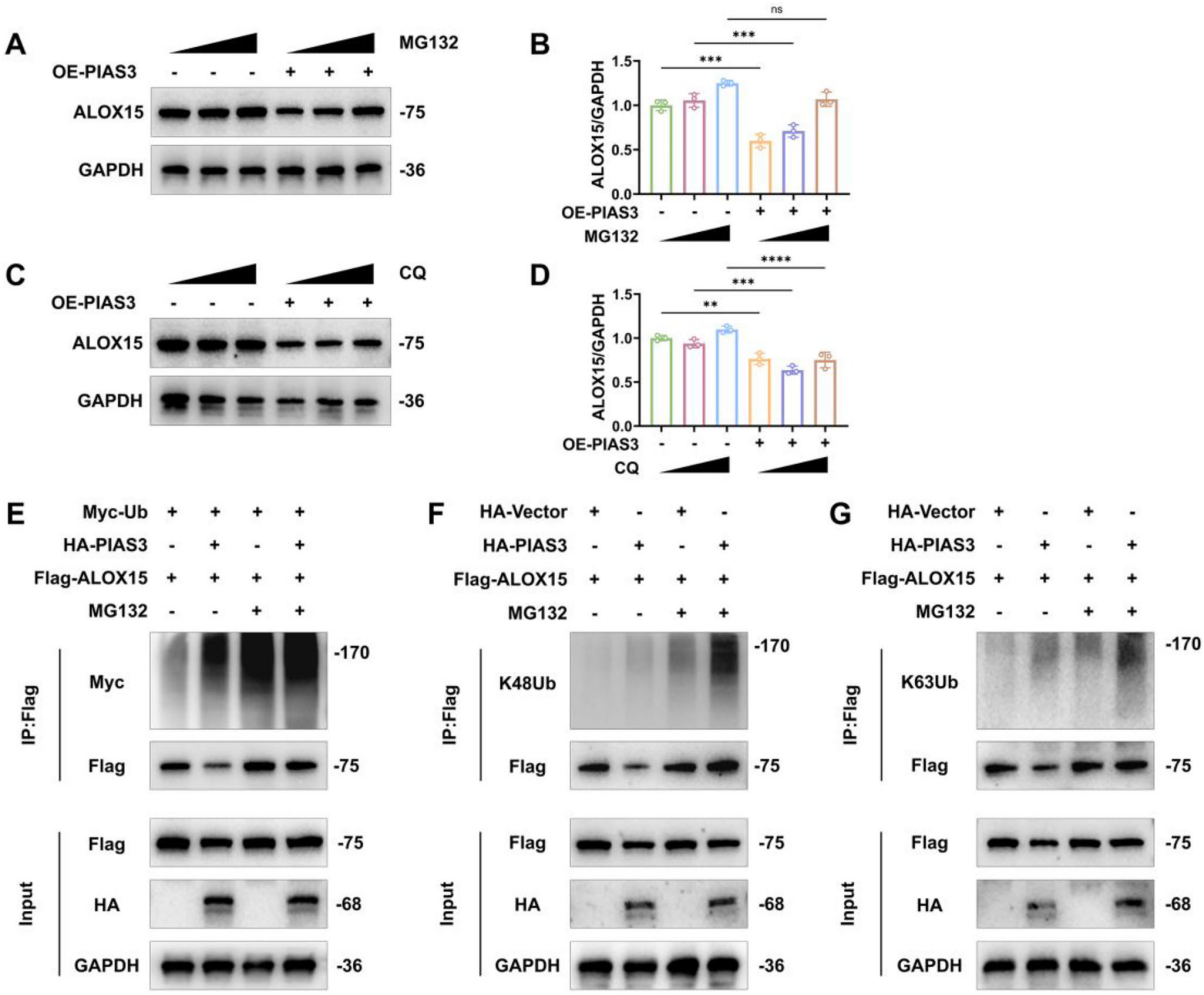
PIAS3 promotes ubiquitination and proteasomal degradation of ALOX15. (A, B) ALOX15 expression after PIAS3 overexpression and increasing concentrations of MG132. (C, D) ALOX15 expression after PIAS3 overexpression and increasing concentrations of CQ. (E–G) Co‐IP analysis of total, K48‐linked, and K63‐linked ubiquitination of Flag‐ALOX15 with or without PIAS3 and MG132. Data are mean ± standard error of the mean (n = 3); one‐way ANOVA; ns, not significant; ^**^ *p* < 0.01, ^***^ *p* < 0.001, ^****^ *p* < 0.0001.

Immunoprecipitation of Flag‐ALOX15 showed that PIAS3 increased total ubiquitination, which accumulated further after MG132 treatment (Figure 4E). PIAS3 preferentially enhanced K48‐linked polyubiquitination of ALOX15, whereas K63‐linked ubiquitination showed little change (Figure 4F,G).

To identify a potential bridge between SUMOylation and ubiquitination, we first focused on RNF4, a canonical SUMO‐targeted ubiquitin ligase (STUbL). Based on transcriptomic alterations in ubiquitination‐related genes and the involvement of E2 enzymes in ubiquitin chain assembly, we additionally examined UBE2G2 as a potential ubiquitination factor. Endogenous co‐IP revealed a clear interaction between ALOX15 and RNF4 but little detectable association with UBE2G2 (Figure S14A,B). After confirming the knockdown efficiencies of *RNF4* and *Ube2g2* by RT‐qPCR (Figure S14C,D), we found that RNF4 knockdown markedly reduced PIAS3‐induced K48‐linked ubiquitination of ALOX15 and prevented the associated reduction in ALOX15 abundance, whereas UBE2G2 knockdown had little effect (Figure S14E). Molecular docking further supported structurally compatible ALOX15–RNF4 interactions in both rat and human proteins (Figures S15 and S16). These findings suggest that RNF4 may serve as an important molecular bridge linking PIAS3‐dependent ALOX15 SUMOylation to downstream K48‐linked ubiquitination.

### 2.7 Fabrication and Characterization of the OLNPs@Gel Delivery Platform

To restore PIAS3 expression locally, OA‐C12 was synthesized and structurally characterized (Figure 5A and Figure S17), and PIAS3 circRNA was subsequently formulated into OA‐C12‐based LNPs using microfluidic mixing (Figure 5B). Sixteen formulations with different molar ratios of OA‐C12, 1,2‐dimyristoyl‐rac‐glycero‐3‐methoxypolyethylene glycol‐2000 (DMG‐PEG2000), 1,2‐dioleoyl‐sn‐glycero‐3‐phosphoethanolamine (DOPE), and cholesterol were screened, and the A11 formulation was selected based on its transfection efficiency in OCCM‐30 cells (Figure 5C and Figure S18). The optimized OLNPs were subsequently incorporated into a photocrosslinkable F127DA hydrogel containing lithium phenyl‐2,4,6‐trimethylbenzoylphosphinate (LAP) and crosslinked with 405‐nm light to generate OLNPs@Gel (Figure 5D).

**FIGURE 5:**
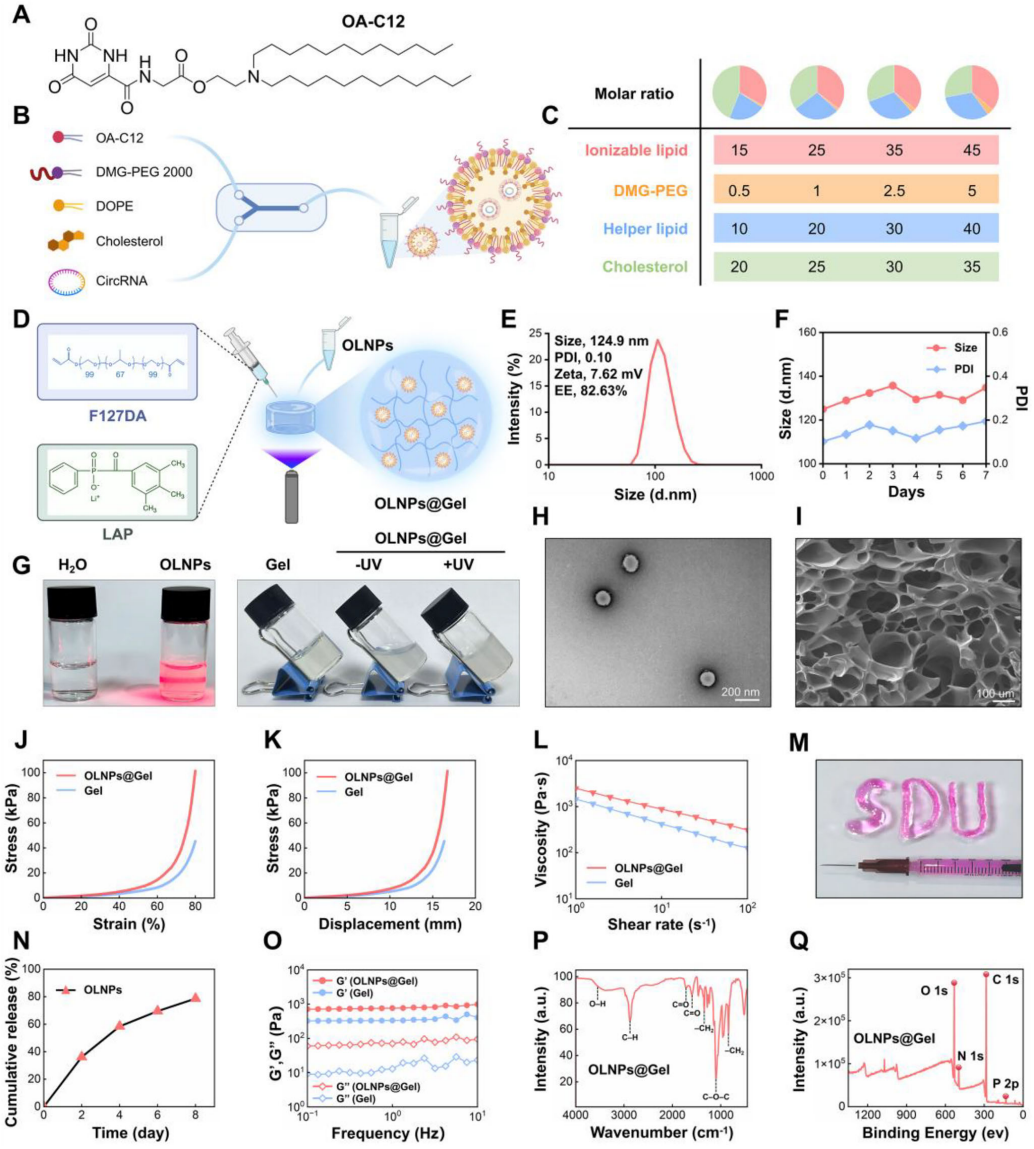
Fabrication and physicochemical characterization of OLNPs and OLNPs@Gel. (A) Chemical structure of OA‐C12. (B) Microfluidic assembly of circRNA‐loaded OLNPs. (C) Orthogonal formulation design for OA‐C12, DMG‐PEG2000, DOPE, and cholesterol. (D) Incorporation of OLNPs into photocrosslinked F127DA hydrogel. (E) Particle‐size distribution and key physicochemical parameters. (F) Particle size and PDI during 7‐day storage. (G) The Tyndall effect of OLNPs and the solidified state of OLNPs@Gel. (H) Representative TEM image of OLNPs. Scale bar: 200 nm. (I) Representative SEM image of OLNPs@Gel. Scale bar: 100 µm. (J, K) Stress–strain and stress–displacement curves. (L) Shear‐dependent viscosity. (M) Injectability of OLNPs@Gel. (N) Cumulative release of OLNPs from OLNPs@Gel. (O) Frequency scan of the storage modulus (G′) and loss modulus (G″) of Gel and OLNPs@Gel at 37°C. (P) FTIR spectrum. (Q) XPS spectrum.

The optimized OLNPs had an average diameter of 124.9 nm, a polydispersity index (PDI) of 0.10, a zeta potential of 7.62 mV, and a circRNA encapsulation efficiency of 82.63% (Figure 5E). Particle size and PDI remained stable during 7 days of storage (Figure 5F). Upon laser irradiation, a clearly visible light path was observed in the OLNP dispersion, demonstrating a pronounced Tyndall effect and indicating good colloidal dispersibility. Macroscopic imaging showed stable hydrogel formation after photocrosslinking, with F127DA gel without circRNA loading serving as the vehicle hydrogel control (Figure 5G). Transmission electron microscopy (TEM) demonstrated that OLNPs were spherical and well dispersed, whereas scanning electron microscopy (SEM) revealed a porous honeycomb structure of OLNPs@Gel (Figures 5H,I).

Mechanical testing showed increased stress resistance after OLNPs incorporation (Figures 5J,K). Both blank Gel and OLNPs@Gel exhibited shear‐thinning behavior, and the OLNPs@Gel precursor could be readily extruded through a syringe while retaining shape fidelity after injection (Figures 5L,M). Frequency‐sweep rheology showed that the storage modulus remained above the loss modulus across the tested frequency range, confirming a stable gel network, with higher G′ and G″ values after OLNPs loading (Figure 5O).

In vitro release assays demonstrated sustained release of OLNPs from OLNPs@Gel, reaching approximately 80.13% by day 8 (Figure 5N). PIAS3 circRNA was continuously released from PIAS3‐OLNPs@Gel at both pH 6.5 and 7.4 up to day 8, reaching cumulative release of 55.07% and 41.52%, respectively (Figure S19A). Both blank Gel and OLNPs@Gel gradually degraded in artificial saliva, with slightly slower degradation of OLNPs@Gel (Figure S19B). Fourier transform infrared spectroscopy (FTIR) and X‐ray photoelectron spectroscopy (XPS) analyses further confirmed the expected chemical characteristics and elemental composition of the composite hydrogel (Figure 5P,Q). Together, these properties support OLNPs@Gel as an injectable sustained‐release platform for local circRNA delivery.

### 2.8 In Vitro Delivery Performance and Biocompatibility of OLNPs

The intracellular delivery performance of OLNPs was evaluated using Cy5‐labeled circRNA. Confocal microscopy showed minimal intracellular fluorescence after treatment with free Cy5‐circRNA but strong Cy5 signals after OLNPs‐mediated delivery (Figure 6A). Flow cytometry confirmed a marked increase in intracellular Cy5 fluorescence in the Cy5‐circRNA‐OLNPs group, indicating successful delivery (Figure S20A,B).

**FIGURE 6:**
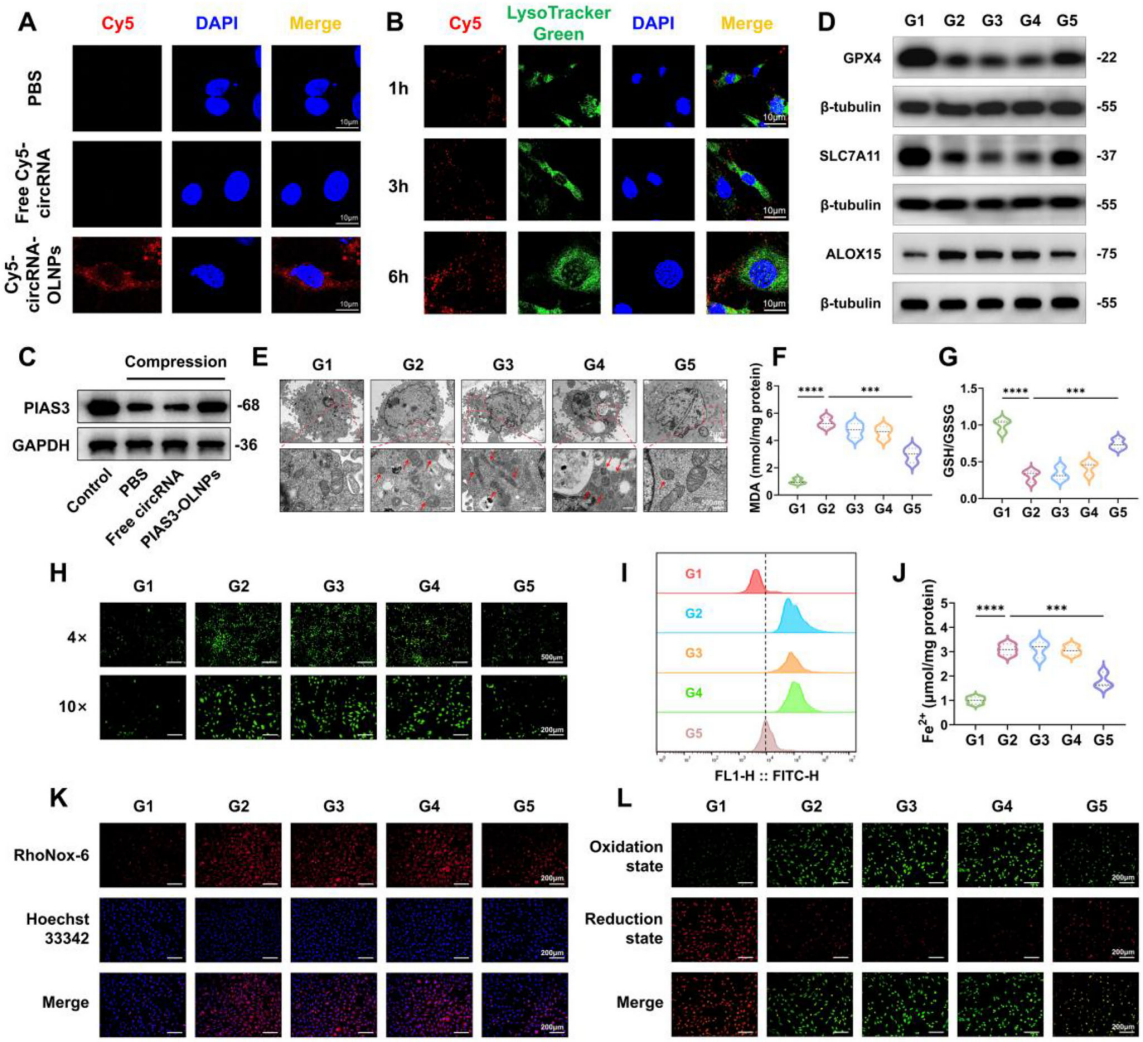
PIAS3‐OLNPs efficiently deliver circRNA and suppress compression‐induced ferroptosis in cementoblasts. (A) Representative confocal images of Cy5‐circRNA uptake. Scale bar: 10 µm. (B) Representative lysosomal escape of Cy5‐circRNA‐OLNPs. Scale bar: 10 µm. (C) PIAS3 expression after compression and circRNA delivery. (D) Western blot of GPX4, SLC7A11, and ALOX15 expression levels. (E) Representative mitochondrial ultrastructure by TEM. Scale bars: 2 µm and 500 nm. (F) MDA content. (G) GSH/GSSG ratio. (H) Representative fluorescence images of intracellular ROS detected using DCFH‐DA. Scale bars: 500 µm (4×) and 200 µm (10×). (I) Flow‐cytometric analysis of intracellular ROS levels. (J) Intracellular Fe^2^^+^ content. (K) Representative fluorescence images of intracellular Fe^2^^+^ detected using RhoNox‐6. Scale bar: 200 µm. (L) Representative fluorescence images of lipid peroxidation detected using BODIPY 581/591 C11, showing oxidized (green) and reduced (red) signals. Scale bar: 200 µm. G1, Control; G2, Compression + PBS; G3, Compression + free circRNA; G4, Compression + blank OLNPs; G5, Compression + PIAS3‐OLNPs. Data are mean ± standard error of the mean (n = 3); one‐way ANOVA; ^***^ *p* < 0.001, ^****^ *p* < 0.0001.

LysoTracker imaging showed substantial colocalization of Cy5‐circRNA with lysosomes at 1 h, reduced colocalization at 3 h, and substantial lysosomal escape by 6 h (Figure 6B). PIAS3‐OLNPs did not significantly reduce cell viability at circRNA concentrations of 100–1250 ng/mL, and live/dead staining similarly showed high cell viability after treatment with blank OLNPs, PIAS3‐OLNPs, or PIAS3‐OLNPs@Gel (Figures S20C and S21). Notably, PIAS3‐OLNPs restored PIAS3 protein expression in compressed OCCM‐30 cells within 24 h (Figure 6C), demonstrating effective functional delivery of PIAS3 circRNA.

### 2.9 PIAS3‐OLNPs Attenuate Compression‐Induced Ferroptosis in Vitro

To determine whether PIAS3 restoration protects cementoblasts from compression‐induced ferroptosis, we examined ferroptosis‐associated proteins, mitochondrial morphology, redox status, iron accumulation, and lipid peroxidation. Compression reduced GPX4 and SLC7A11 expression and increased ALOX15, whereas PIAS3‐OLNPs substantially reversed these changes (Figure 6D and Figure S22A–F).

TEM revealed mitochondrial shrinkage, increased membrane density, and cristae disruption after compression, all of which were largely alleviated by PIAS3‐OLNPs treatment (Figure 6E). Compression also increased MDA and ROS levels and reduced the GSH/GSSG ratio, whereas PIAS3 restoration improved these redox abnormalities (Figure 6F–I). Quantitative Fe^2^^+^ assays and RhoNox‐6 staining demonstrated marked iron accumulation after compression that was reduced by PIAS3‐OLNPs (Figure 6J,K). BODIPY 581/591 C11 staining similarly showed increased lipid oxidation in compressed cells and attenuation after PIAS3 delivery (Figure 6L). These findings indicate that restoration of PIAS3 suppresses multiple biochemical and ultrastructural features of compression‐induced ferroptosis.

### 2.10 PIAS3 Restoration Reduces the Osteoclastogenic Paracrine Activity of Compressed Cementoblasts

To assess the paracrine consequences of cementoblast injury, conditioned media from differently treated OCCM‐30 cells were applied to bone marrow‐derived macrophages (BMDMs) undergoing osteoclast differentiation (Figure 7A). Conditioned medium from compressed cells treated with PBS, free circRNA, or blank OLNPs markedly promoted TRAP‐positive multinucleated osteoclast formation. In contrast, conditioned media from OCCM‐30 cells treated with PIAS3‐OLNPs or Fer‐1 substantially suppressed osteoclastogenesis (Figure 7B). NFATc1, MMP9, CTSK, and ACP5 expression showed corresponding changes (Figure 7C–G).

**FIGURE 7:**
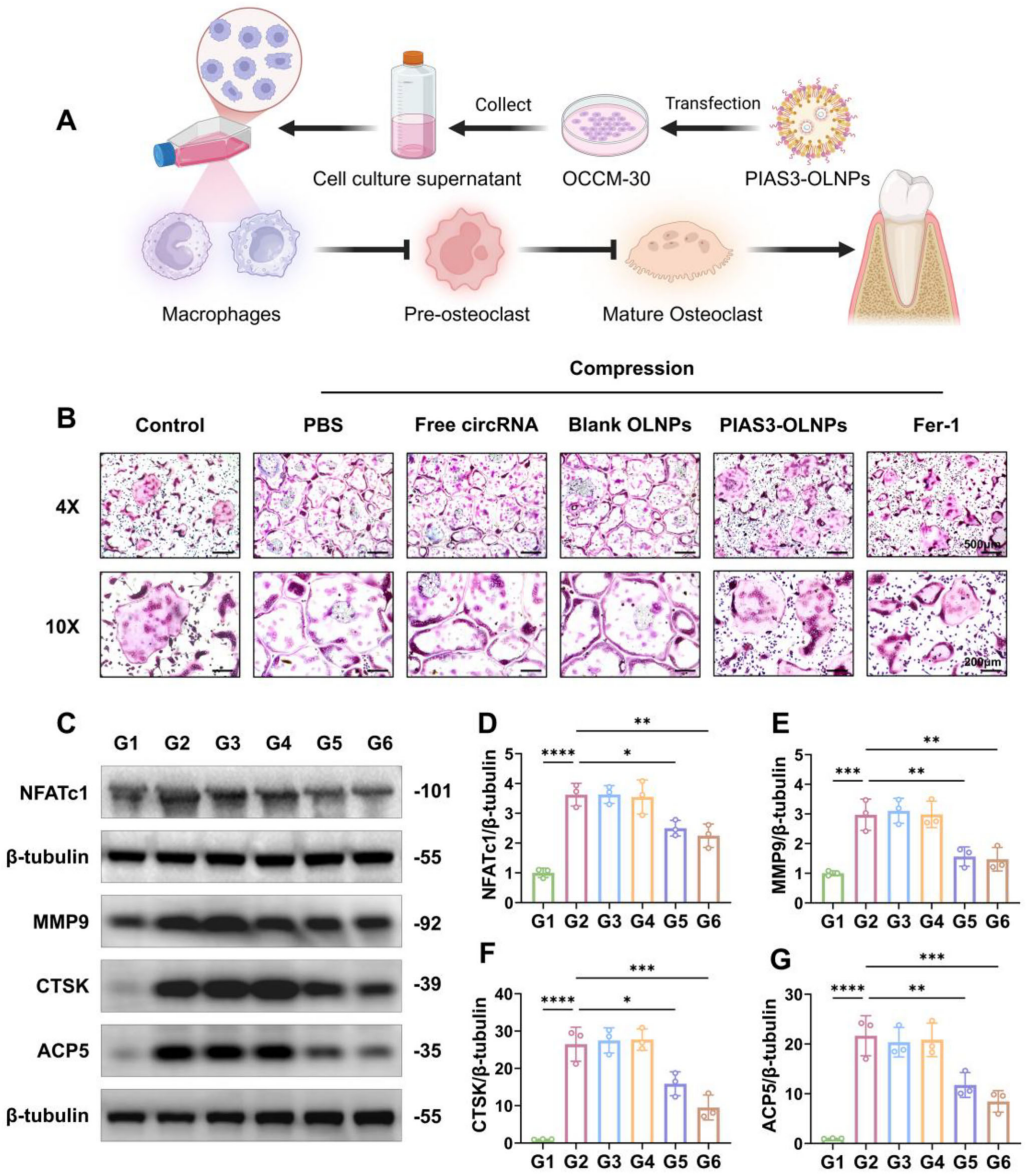
PIAS3‐OLNPs suppress osteoclastogenesis induced by conditioned medium from compressed cementoblasts. (A) Experimental workflow for conditioned‐medium‐driven osteoclast differentiation. (B) Representative TRAP‐stained images of osteoclasts. Scale bars: 500 µm for 4× images and 200 µm for 10× images. (C) Western blot analysis of the osteoclast‐related proteins NFATc1, MMP9, CTSK, and ACP5. (D–G) Densitometric quantification of NFATc1, MMP9, CTSK, and ACP5, respectively. G1, Control; G2, Compression + PBS; G3, Compression + free circRNA; G4, Compression + blank OLNPs; G5, Compression + PIAS3‐OLNPs; G6, Compression + Fer‐1. Data are mean ± standard error of the mean (n = 3); one‐way ANOVA; ^*^ *p* < 0.05, ^**^ *p* < 0.01, ^***^ *p* < 0.001, ^****^ *p* < 0.0001.

Enzyme‐linked immunosorbent assay (ELISA) showed that compression decreased OPG while increasing RANKL and M‐CSF in OCCM‐30‐derived conditioned medium. These alterations were partially reversed by both PIAS3‐OLNPs and Fer‐1 (Figure S23A–C). RhoNox‐6 staining further showed reduced Fe^2^^+^‐associated fluorescence in BMDMs exposed to conditioned medium from the PIAS3‐OLNPs and Fer‐1 groups (Figure S23D). To assess potential OLNPs carryover, BMDMs were directly exposed to either Cy5‐circRNA‐OLNPs or PIAS3‐OLNPs, or treated with conditioned medium from OCCM‐30 cells exposed to the corresponding OLNPs formulation. Direct Cy5‐circRNA‐OLNPs and PIAS3‐OLNPs exposure markedly increased Cy5 fluorescence and PIAS3 expression, whereas conditioned medium produced neither a detectable increase in Cy5 signal nor significant PIAS3 upregulation (Figure S24A–C). Thus, although trace PIAS3‐OLNPs carryover cannot be excluded, it was insufficient to induce PIAS3 overexpression in BMDMs. Together, these findings support that PIAS3 restoration reduces the osteoclastogenic paracrine activity of compressed cementoblasts at least in part through inhibition of cementoblast ferroptosis, as supported by the comparable suppressive effects of PIAS3‐OLNPs and Fer‐1. However, the present data do not exclude direct PIAS3‐dependent regulation of RANKL, OPG, or M‐CSF, or contributions from other ferroptosis‐independent mechanisms. A functionally relevant direct effect of residual PIAS3‐OLNPs on BMDMs appears unlikely based on the carryover experiments.

### 2.11 In Vivo Biosafety and Local Retention

Hematoxylin and eosin (H&E) staining of the heart, liver, spleen, lung, and kidney showed no obvious histopathological abnormalities after treatment with the OLNP‐based formulations (Figure S25A), supporting the favorable biosafety of PIAS3‐OLNPs@Gel. In vivo fluorescence imaging showed that both DiR‐labeled OLNPs and OLNPs@Gel were initially retained at the administration site, but the signal from free OLNPs declined more rapidly. In contrast, OLNPs@Gel exhibited stronger and more persistent local fluorescence for up to 72 h (Figure S25B), indicating prolonged local retention of the hydrogel formulation.

### 2.12 PIAS3‐OLNPs@Gel Suppress Ferroptosis and Attenuate OIRR In Vivo

The therapeutic efficacy of local PIAS3 delivery was evaluated in rats treated with PBS, blank OLNPs@Gel, PIAS3‐OLNPs, or PIAS3‐OLNPs@Gel. Micro‐CT showed extensive pressure‐side root resorption in the OIRR + PBS group, whereas blank OLNPs@Gel provided little protection. Both PIAS3‐OLNPs and PIAS3‐OLNPs@Gel reduced the number and severity of resorption lacunae, with the hydrogel formulation providing more root surface protection (Figure 8A,B). Tooth movement distance remained comparable between the OIRR and treatment groups (Figure S26A,B).

**FIGURE 8:**
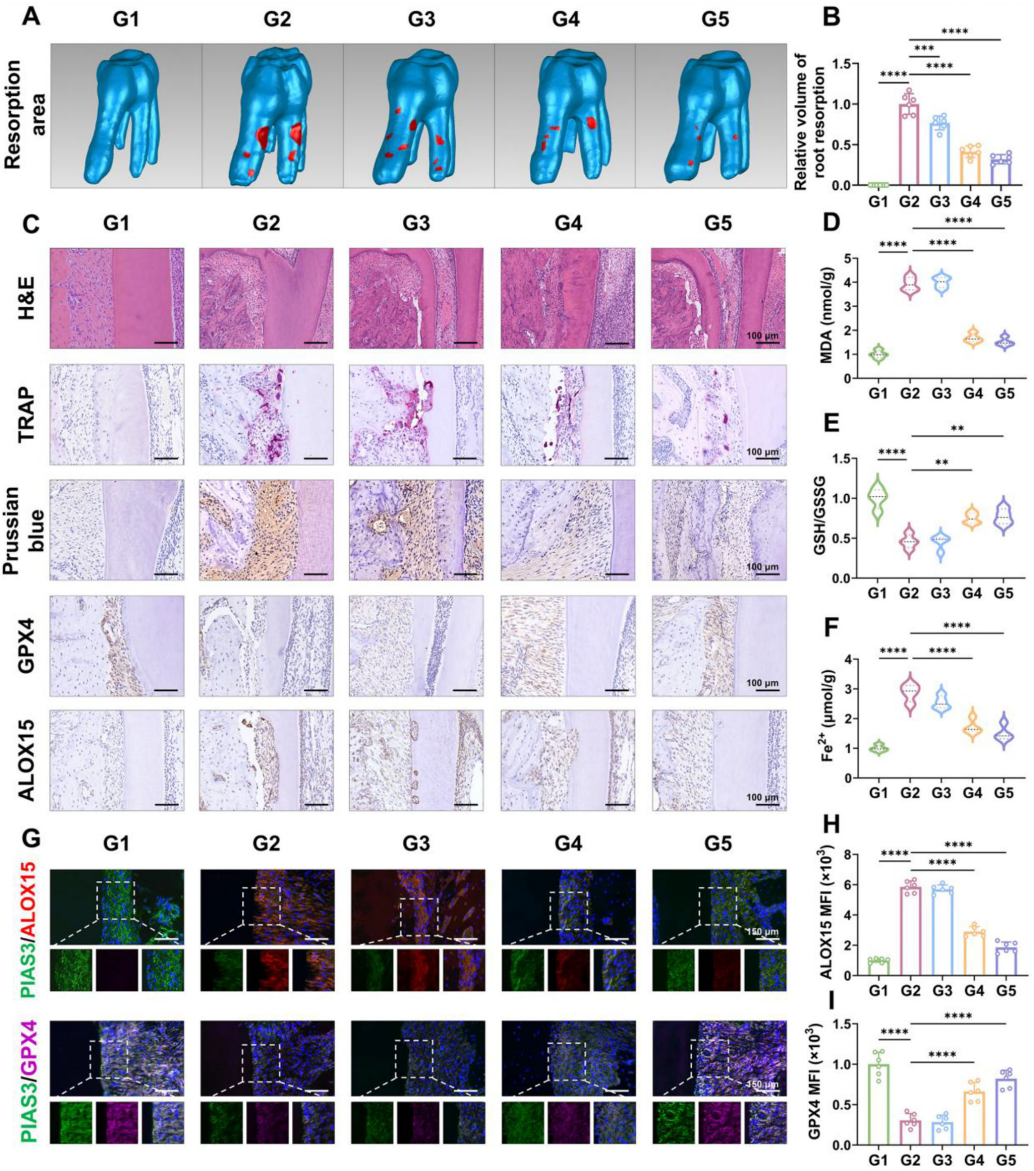
Local PIAS3 circRNA delivery attenuates ferroptosis and OIRR in vivo. (A) Representative micro‐CT reconstructions of maxillary first‐molar roots; resorption lesions are highlighted in red. (B) Quantification of the relative root resorption volume. (C) Representative images of H&E, TRAP, Prussian blue, and immunohistochemical staining for GPX4 and ALOX15 in periodontal tissues on the compression side. Scale bar: 100 µm. (D–F) Periodontal tissue levels of MDA, the GSH/GSSG ratio, and Fe^2^^+^, respectively. (G) Representative dual‐immunofluorescence images for PIAS3 (green) and ALOX15 (red), or PIAS3 (green) and GPX4 (magenta); nuclei were counterstained with DAPI (blue). Dashed boxes indicate the regions shown at higher magnification below. Scale bar: 150 µm. (H, I) Quantification of ALOX15 and GPX4 mean fluorescence intensity (MFI), respectively. G1, Control; G2, OIRR + PBS; G3, OIRR + blank OLNPs@Gel; G4, OIRR + PIAS3‐OLNPs; G5, OIRR + PIAS3‐OLNPs@Gel. Data are presented as mean ± standard error of the mean (n = 6 independent rats per group); one‐way ANOVA; ^**^ *p* < 0.01, ^***^ *p* < 0.001, ^****^ *p* < 0.0001.

Histological analysis confirmed severe root‐surface defects and periodontal ligament disorganization in the OIRR + PBS and blank OLNPs@Gel groups, accompanied by abundant TRAP‐positive osteoclast‐like cells. These changes were substantially attenuated after treatment with PIAS3‐OLNPs or PIAS3‐OLNPs@Gel (Figure 8C). Prussian blue staining demonstrated increased peri‐root iron deposition during OIRR, whereas PIAS3 delivery reduced iron accumulation. OIRR also decreased GPX4 and increased ALOX15 in periodontal tissues; both changes were attenuated by the PIAS3 formulations but not by blank OLNPs@Gel (Figure 8C). Consistently, PIAS3 treatment reduced MDA and Fe^2^^+^ levels and partially restored the GSH/GSSG ratio (Figure 8D–F).

Dual immunofluorescence further showed weak PIAS3 and GPX4 signals and prominent ALOX15 fluorescence in OIRR + PBS and blank OLNPs@Gel tissues. PIAS3 circRNA delivery restored PIAS3 expression, reduced ALOX15, and increased GPX4, with the most pronounced changes in the PIAS3‐OLNPs@Gel group (Figure 8G–I).

Cementoblast‐enriched primary cells isolated from Control, OIRR + PBS, and OIRR + PIAS3‐OLNPs@Gel rats were analyzed to provide supporting evidence. OIRR decreased PIAS3 and GPX4 and increased ALOX15 and ACSL4 levels, whereas PIAS3‐OLNPs@Gel partially reversed these changes (Figure S27A,B). Treatment also reduced lipid peroxidation, Fe^2^^+^ accumulation, and MDA production and increased the GSH/GSSG ratio (Figure S27C–F), while restoring OIRR‐reduced ALOX15 SUMOylation and K48‐linked ubiquitination (Figure S28A,B). These findings support an association between in vivo PIAS3 circRNA treatment and restoration of the PIAS3–ALOX15 SUMOylation–ubiquitination axis in cementoblast‐enriched primary cells.

## 3 Discussion

OIRR is a frequent and largely irreversible complication of excessive orthodontic compression, yet the mechanisms linking mechanical stress to root damage remain incompletely understood [24]. Here, we identify cementoblast ferroptosis as an important component of OIRR and define a PIAS3–ALOX15 regulatory axis linking compression to lipid peroxidation. Compression reduced PIAS3, impaired ALOX15 SUMOylation and K48‐linked ubiquitination, and promoted ALOX15 accumulation, ferroptotic injury, osteoclastogenesis, and root resorption. Local delivery of PIAS3 circRNA using OA‐C12‐based LNPs embedded in F127DA hydrogel attenuated ferroptosis‐associated tissue changes and root resorption in vivo, with analyses of cementoblast‐enriched primary cells supporting restoration of the PIAS3–ALOX15 regulatory axis.

Previous studies have implicated ferroptosis in OIRR, including NCOA4/FTH1‐dependent ferritinophagy in periodontal ligament cells and cGAS–STING/GPX4 signaling in cementoblasts [9, 12]. Our results extend these observations with supporting evidence from cementoblast‐enriched primary cells isolated from rats. These cells showed increased ACSL4, lipid oxidation, MDA, and Fe^2^^+^ accumulation together with reduced SLC7A11, FTH1, and GSH/GSSG, and these changes were reversed by Fer‐1. Although bulk periodontal RNA‐seq cannot assign transcriptional changes to individual cell populations, the increase in ALOX15 and decrease in PIAS3 were independently confirmed in the cementoblast‐enriched primary cell population. Conditioned‐medium experiments further showed that compressed cementoblasts enhanced osteoclast differentiation, whereas both PIAS3 restoration and Fer‐1 suppressed this response, supporting the involvement of ferroptosis in this paracrine effect [25]. Importantly, these findings do not establish that the anti‐osteoclastogenic effect of PIAS3 restoration is exclusively ferroptosis‐dependent. PIAS3‐OLNPs partially changed RANKL, OPG, and M‐CSF levels in cementoblast‐derived conditioned medium. However, the present experiments cannot distinguish whether these changes occur secondary to ferroptosis suppression or reflect direct PIAS3‐dependent regulation of these osteoclastogenic factors. Contributions from other ferroptosis‐independent pathways also cannot be excluded. Notably, we acknowledge that trace amounts of residual PIAS3‐OLNPs may remain in the conditioned medium. Conditioned medium from PIAS3‐OLNPs‐treated OCCM‐30 cells did not increase PIAS3 protein expression in BMDMs, indicating that residual PIAS3‐OLNPs were present at concentrations below the threshold required for effective PIAS3 delivery and were therefore unlikely to exert direct biological effects on BMDMs.

Mechanistically, ALOX15 accumulation reflects both transcriptional upregulation and impaired PIAS3‐mediated degradation. Although PIAS3 did not measurably affect *Alox15* transcription, it regulated ALOX15 protein stability through SUMOylation‐dependent turnover. Thus, transcriptional upregulation and impaired PIAS3‐mediated degradation jointly contribute to ALOX15 accumulation, although their relative contributions cannot be resolved from the present data. Given the established role of ALOX15 in PUFA‐phospholipid peroxidation, its sustained accumulation may amplify ferroptotic injury [26]. PIAS3 promoted SUMO3 modification at Lys50, and K50R reduced SUMOylation, prolonged ALOX15 half‐life, and weakened PIAS3‐dependent degradation, consistent with the context‐dependent regulation of protein stability by SUMOylation and other post‐translational modifications [27, 28]. SENP1, SENP2, SENP3, and SENP5 protein levels were unchanged after compression, arguing against altered abundance of these deSUMOylases as the major explanation, although changes in enzymatic activity cannot be excluded. Importantly, RNF4 interacted with ALOX15, and RNF4 knockdown reduced PIAS3‐induced K48‐linked ubiquitination and prevented ALOX15 degradation, supporting RNF4 as a key molecular link between SUMOylation and ubiquitination, consistent with the established role of STUbLs [29]. UBE2G2 was examined as a downstream ubiquitination component based on its role in ubiquitination and OIRR‐associated transcriptomic changes. However, its weak association with ALOX15 and limited effect on ubiquitination suggest little effect on the PIAS3‐dependent degradation of ALOX15. Consistently, ex vivo analysis of cementoblast‐enriched primary cells isolated from OIRR rats showed reduced ALOX15 SUMOylation and K48‐linked ubiquitination, both of which increased in cells isolated after in vivo PIAS3‐OLNPs@Gel treatment. These findings provide ex vivo support rather than direct cell‐specific in vivo evidence.

The therapeutic significance of this study lies in combining pathway specificity with localized delivery. Circular RNA offers the potential for sustained protein expression [30], while incorporation of PIAS3‐OLNPs into F127DA hydrogel prolonged local retention and sustained release of both OLNPs and circRNA. Blank OLNPs@Gel provided little protection, whereas PIAS3‐containing formulations reduced ferroptosis and root resorption without significantly altering orthodontic tooth movement distance. These findings support the use of injectable hydrogel‐based gene therapy as a novel strategy to alleviate OIRR.

Several limitations remain. Current cell and rat models cannot fully recapitulate human periodontal biomechanics and the intraoral microenvironment. Future studies using conditional knockout animal models or cementoblast‐targeted gene editing could provide stronger cell‐specific causal evidence. Validation in primary human cementoblasts and periodontal organoids, together with long‐term evaluation of circRNA immunogenicity, hydrogel safety, and treatment durability, will be important for clinical translation.

## 4 Materials and Methods

### 4.1 Animals

Male Sprague–Dawley (SD) rats (6–8 weeks old, weighing 180–310 g) and male C57BL/6 mice (6–8 weeks old, weighing 18–25 g) were purchased from Beijing Vital River Laboratory Animal Technology Co., Ltd. (China). With a 12‐h light/dark cycle, a temperature of 22°C–24°C, a relative humidity of 40–60%, standard food, and unlimited access to water, the animals were housed under specific pathogen‐free conditions. SD rats were chosen for in vivo experiments. BMDMs were isolated from C57BL/6 mice. All animal experiments were approved by the Ethical Review Committee for Experimental Animal Welfare of the School and Hospital of Stomatology, Shandong University, Jinan, China (Approval No. 20260504) and were conducted in accordance with the Basel Declaration.

In the ferroptosis inhibition experiment, the rats were divided into Control, OIRR, and OIRR + Fer‐1 groups (n = 6 independent rats per group). In the PIAS3 delivery experiment, the rats were divided into Control, OIRR + PBS, OIRR + blank OLNPs@Gel, OIRR + PIAS3‐OLNPs, and OIRR + PIAS3‐OLNPs@Gel groups (n = 6 independent rats per group). Each rat constituted a biological replicate. Under inhalation anesthesia with isoflurane, a nickel‐titanium spring was secured to the maxillary first molar using a 0.25‐mm orthodontic ligature wire, with the opposite end anchored to the maxillary incisors. The mesial surface of the maxillary first molar and the cervical region of the maxillary incisors were lightly abraded to ensure mechanical retention, followed by fixation using light‐cured composite resin. The appliance delivered a continuous mesial traction force of approximately 50 g. Appliance integrity and force magnitude were assessed every 2 days. Rats with inappropriate force levels or more than two episodes of appliance dislodgement were excluded from the statistical analysis. In the Fer‐1 group, 10 µL of a 10 mm Fer‐1 solution was locally injected into the mesial gingival sulcus. In the OIRR + PIAS3‐OLNPs group, 10 µL of PIAS3‐OLNPs suspension was injected into the mesial gingival sulcus using the same administration schedule. In the OIRR + blank OLNPs@Gel and OIRR + PIAS3‐OLNPs@Gel groups, 10 µL of the composite hydrogel was injected into the mesial gingival sulcus of the maxillary first molar with a microsyringe, followed by light curing to ensure sufficient penetration along the root surface. To prevent dislodgement of the drugs, a thin layer of sterile petrolatum was applied to the gingival margin after each administration. In the ferroptosis inhibition experiment, the Control group and OIRR group were injected with the same volume of DMSO instead of Fer‐1 and coated with sterile petrolatum. In the PIAS3 delivery experiment, the Control and OIRR + PBS groups were injected with an equal volume of PBS in place of the drug and coated with sterile petrolatum. Local treatment was administered once every 3 days for 14 days, for a total of five administrations. The route and dosage of local administration were determined on the basis of the anatomical constraints of the gingival sulcus and previously published protocols [31, 32].

For analysis of the PIAS3–ALOX15 post‐translational regulatory axis following in vivo treatment, additional rats were assigned to the Control, OIRR + PBS, and OIRR + PIAS3‐OLNPs@Gel groups (n = 6 independent rats per group). Orthodontic force application and local PIAS3‐OLNPs@Gel administration were performed according to the same procedures and treatment schedule described above. Cementoblast‐enriched primary cells were subsequently isolated for ALOX15 immunoprecipitation followed by SUMOylation and ubiquitination analyses.

### 4.2 Cell Culture

OCCM‐30 cells were purchased from Guangzhou Jennio Biotech Co., Ltd., and cultured in high‐glucose Dulbecco's modified Eagle's medium (DMEM) supplemented with 10% fetal bovine serum (FBS), 100 U/mL penicillin, and 100 µg/mL streptomycin. Cells at passages 3–5 were employed in the subsequent experiments.

Cementoblast‐enriched primary cells were isolated using a previously described sequential enzymatic digestion protocol [33]. Briefly, first molars were harvested immediately after sacrifice and transferred into pre‐chilled PBS. After removal of gross periodontal connective tissue, the root surfaces were subjected to sequential digestion with 2 mg/mL type I collagenase and 0.25% trypsin at 37°C, with released cells collected at 30‐min intervals. Based on the previously validated enrichment of cementoblasts in the late root surface digestion fraction, cells collected at 120 min were designated as the cementoblast‐enriched population. Cementoblast enrichment was further assessed by immunofluorescence staining for BSP and CD146. Cells at passages 3–5 were used for experiments.

### 4.3 In Vitro Mechanical Loading

OCCM‐30 cells were subjected to static compression according to previously established protocols to model a defined cementoblast‐intrinsic response to compression‐side mechanical stress during orthodontic tooth movement [34, 35]. Briefly, sterile cover glasses and glass bottles containing steel balls were placed on the cells. The pressure was adjusted to 2.0 g/cm^2^. Uncompressed cells served as the Control group, while cells subjected to compression received the indicated treatments. For the Fer‐1 group, a 10 mm stock solution of Fer‐1 was diluted in PBS to a final working concentration of 2 µm [9]. During time‐course assays, cells were harvested at 0, 6, 12, 24, and 48 h after compression loading. A period of 24 h was adopted in the subsequent intervention experiments. The culture supernatants were collected and centrifuged at 1006 × g for 10 min to remove cell debris. The resulting supernatants were harvested for subsequent experiments.

### 4.4 RNA‐seq Analysis

Periodontal tissues surrounding the tooth roots on the compression side were collected from the Control and OIRR groups (n = 6 independent rats per group) and lysed using TRIzol reagent (Invitrogen, USA) for total RNA extraction. RNA purity and concentration were assessed using a NanoDrop 2000 spectrophotometer (Thermo Scientific, USA), and RNA integrity was evaluated with an Agilent 2100 Bioanalyzer. Only RNA samples with an A260/A280 ratio of 1.8–2.1, an A260/A230 ratio ≥ 2.0, and an RNA integrity number (RIN) ≥ 7.0 were used for subsequent library construction.

cDNA libraries were prepared using the VAHTS Universal V10 RNA‐seq Library Preparation Kit (Vazyme, China) according to the manufacturer's instructions. The libraries were subsequently sequenced on an Illumina NovaSeq platform to generate 150‐bp paired‐end reads. Raw reads were processed using fastp to remove adapters and low‐quality reads, and the resulting clean reads were aligned to the reference genome using HISAT2.

Gene expression levels were estimated as fragments per kilobase of transcript per million mapped reads (FPKM), and gene‐level read counts were generated using HTSeq. Differentially expressed genes (DEGs) were identified using DESeq2 on the basis of raw read counts, with thresholds of |log_2_(fold change)| > 1 and q‐value < 0.05. The raw RNA‐seq data generated in this study have been deposited in the NCBI Gene Expression Omnibus (GEO) under accession number GSE347094.

### 4.5 Untargeted Metabolomics

Periodontal tissues collected from the compression side of tooth roots (n = 6 independent rats per group) were homogenized in an ice‐cold methanol/water mixture (4:1, v/v) containing L‐2‐chlorophenylalanine as the internal standard. Following cryogenic grinding, ice‐bath ultrasonication, and incubation at −40°C, samples were centrifuged at 12 000 rpm for 15 min at 4°C. The supernatants were vacuum‐dried, reconstituted in methanol/water (1:4, v/v), and stored at −80°C until analysis. Quality control samples were prepared by pooling equal aliquots from all individual samples. Untargeted metabolomic profiling was performed using ultra‐performance liquid chromatography (UPLC) coupled with high‐resolution mass spectrometry in both positive and negative electrospray ionization modes. Raw data were processed for peak detection, alignment, and normalization, and metabolite identification was performed by matching against HMDB, METLIN, and LIPID MAPS databases. Multivariate analyses, including PCA, PLS‐DA, and OPLS‐DA, were conducted to evaluate metabolic differences among groups. False discovery rate (FDR)‐adjusted *p‐values* were reported as q‐values. Differential metabolites were screened based on |log_2_(fold change)| > 1, q < 0.05, and variable importance in projection (VIP) > 1. The raw untargeted metabolomics data generated in this study have been deposited in the OMIX database of the National Genomics Data Center (NGDC), China National Center for Bioinformation (CNCB), under accession number OMIX020579.

### 4.6 RT‐qPCR

In tissue RT‐qPCR analysis, periodontal tissues were harvested from individual rats, with each rat serving as one biological replicate (n = 6 independent rats per group). In cellular RT‐qPCR analysis, three independent biological experiments were performed using OCCM‐30 cells seeded in separate batches (n = 3). For RT‐qPCR analysis of cementoblast‐enriched primary cells, cells isolated from individual rats were treated as independent biological replicates (n = 6 independent rats per group). Total RNA was extracted from periodontal tissues and cells using the SteadyPure Quick RNA Extraction Kit (Accurate Biotechnology Co., Ltd., China). An appropriate amount of RNA was reverse‐transcribed into cDNA using the Evo M‐MLV RT Mix Kit with gDNA Clean for RT‐qPCR Ver. 2 (Accurate Biotechnology Co., Ltd., China). RT‐qPCR was conducted using the SYBR Green Premix Pro Taq HS qPCR Kit (Accurate Biotechnology Co., Ltd., China). The primer sequences for the target genes are provided in Table S1. Relative mRNA expression levels were calculated using the 2^−ΔΔCt^ method.

### 4.7 Western Blotting

Protein was extracted from OCCM‐30 cells or cementoblast‐enriched primary cells in RIPA lysis buffer (Solarbio, China) supplemented with protease and phosphatase inhibitors at 4°C. Protein concentrations were determined with a BCA Assay Kit (Beyotime, China). Equal amounts of protein were separated through sodium dodecyl sulfate–polyacrylamide gel electrophoresis (SDS‐PAGE) and transferred onto 0.45‐µm polyvinylidene fluoride (PVDF) membranes (Millipore, USA). The membranes were subsequently blocked with 5% nonfat milk for 2 h, followed by overnight incubation at 4°C with anti‐GPX4 (125 066; Abcam, UK), anti‐SLC7A11 (307 601; Abcam, UK), anti‐ALOX15 (244205; Abcam, UK), anti‐PIAS3 (13 486; Proteintech, USA), anti‐ACSL4 (22401; Proteintech, USA), anti‐FTH1 (11682; Proteintech, USA), anti‐SENP1 (25349; Proteintech, USA), anti‐SENP2 (29772; Proteintech, USA), anti‐SENP3 (17659; Proteintech, USA), anti‐SENP5 (19529; Proteintech, USA), anti‐RNF4 (17810; Proteintech, USA), anti‐UBE2G2 (10722; Proteintech, USA), anti‐SUMO2/3 (11251; Proteintech, USA), anti‐Flag tag (0053; Abmart, China), anti‐HA tag (3724; CST, USA), anti‐His tag (12698; CST, USA), anti‐Myc tag (2276; CST, USA), anti‐ubiquitin (10201; Proteintech, USA), anti‐K48 ubiquitin (18661; UpingBio, China), anti‐K63 ubiquitin (19058; UpingBio, China), anti‐NFATc1 (1539; ABclonal, USA), anti‐MMP9 (27306; Proteintech, USA), anti‐CTSK (11239; Proteintech, USA), anti‐ACP5 (11594; Proteintech, USA), anti‐GAPDH (1210; HUABIO, China), and anti‐β‐tubulin (20005; Abmart, China) primary antibodies. Afterward, the membranes were incubated with HRP‐conjugated secondary antibodies (0001; Affinity Biosciences, China) for 1 h, followed by imaging with ECL reagent (201; EpiZyme, China). The protein band densities were analyzed in ImageJ. The relative expression of each target protein was compared with that of the corresponding reference protein and standardized.

### 4.8 Synthesis and Characterization of OLNPs

PIAS3‐circRNA, EGFP‐circRNA, and Cy5‐circRNA were purchased from GenScript Biotech Corporation. OLNPs were prepared by mixing lipids with PIAS3 circRNA using a microfluidic system (INano L, Micro & Nano (Shanghai) Biologics Co., Ltd.). Briefly, OA‐C12, DMG‐PEG2000, DOPE, and cholesterol were dissolved in ethanol as the organic phase. PIAS3 circRNA was dissolved in a citrate buffer (pH 4.0) as the aqueous phase at a lipid‐to‐RNA mass ratio of 10:1. The resulting mixture was dialyzed against RNase‐free PBS (pH 7.4) to remove ethanol, followed by concentration through ultrafiltration. All the OLNPs were stored at 4°C. The particle size, PDI, and zeta potential of the OLNPs were measured through dynamic light scattering (DLS). The encapsulation efficiency was determined using the Quant‐iT RiboGreen assay (Thermo Fisher Scientific, USA). The morphological features were observed through TEM (HT7800, Hitachi, Japan).

### 4.9 Synthesis and Characterization of the F127DA Hydrogel and OLNPs@Gel

F127DA hydrogel was synthesized according to the literature [36, 37]. Briefly, Pluronic F127 was dissolved in anhydrous dichloromethane under magnetic stirring, followed by the slow addition of triethylamine and acryloyl chloride under ice‐bath conditions. The reaction was maintained at 4°C for 1 h and then allowed to proceed at room temperature for 24 h. The precipitated triethylamine salt was removed through filtration, and the filtrate was precipitated into ice‐cold anhydrous diethyl ether. The obtained F127DA was frozen at −20°C for 24 h and lyophilized at −55°C for 48 h. To prepare the precursor solution, lyophilized F127DA was dissolved at a concentration of 5% (w/v) in a 0.5% (w/v) lithium acylphosphinate photoinitiator solution at 4°C until complete dissolution was achieved. To prepare OLNPs@Gel, OLNPs were dispersed in sterile PBS, gently sonicated to obtain a homogeneous suspension, and then added dropwise to the precooled F127DA precursor solution under mixing at 4°C. The final OLNP concentration was adjusted to 1 µg/mL. The solution was filtered through a 0.22‐µm filter to remove bacteria. The resulting precursor solution was subsequently transferred into a sterile mold and photocrosslinked under 405‐nm light irradiation at an intensity of 30 mW cm^−^^2^ for 30 s to form the OLNPs@Gel composite hydrogel. Blank 5% F127DA hydrogels were prepared using the same procedure but without the use of OLNPs. All the hydrogels were subsequently washed with sterile PBS to remove residual soluble components.

The internal porous structure of OLNPs@Gel was observed through SEM. The mechanical properties of OLNPs@Gel were evaluated using a universal testing machine. The shear‐dependent viscosity of the OLNPs@Gel precursor solution was measured with a rotational rheometer. The injectability of OLNPs@Gel was assessed by extrusion through a syringe needle. The in vitro release profile of OLNPs from OLNPs@Gel was determined by immersing OLNPs@Gel in PBS at 37°C and collecting the supernatant at predetermined time points. For PIAS3 circRNA release, PIAS3‐OLNPs@Gel was incubated in RNase‐free PBS at pH 6.5 or 7.4 at 37°C for 8 days. At predetermined time points, the release medium was collected and replenished with an equal volume of fresh buffer. The cumulative release of PIAS3 circRNA was quantified using the Quant‐iT RiboGreen RNA Assay Kit (Thermo Fisher Scientific, USA). The viscoelastic properties of the blank Gel and OLNPs@Gel were characterized through oscillatory rheological analysis, and the storage modulus (G′) and loss modulus (G″) were recorded as functions of frequency. The chemical structure of OLNPs@Gel was analyzed through FTIR spectroscopy. The surface elemental composition and chemical states of OLNPs@Gel were determined through XPS.

### 4.10 OCCM‐30 Transfection and Cytotoxicity Assays

OCCM‐30 cells were transfected with OLNPs at a concentration of 1 µg/mL. Referring to the method of preparing hydrogel extracts in the published literature, sterile OLNPs@Gel samples were incubated in complete medium at a wet gel‐to‐medium ratio of 0.1 g/mL at 37°C under gentle agitation [38]. The extracts were collected aseptically and centrifuged at low speed to remove visible hydrogel debris. OCCM‐30 cells were exposed to extracts for 24 h. The extracts from blank F127DA hydrogels were used as carrier controls. The OLNP concentration in the extract was adjusted to 1 µg/mL before application to OCCM‐30 cells.

To evaluate the in vitro transfection efficiency, OLNPs encapsulated with EGFP circRNA were added. Transfection was assessed through fluorescence microscopy and flow cytometry after 24 h of incubation.

In the cytotoxicity assays, OCCM‐30 cells were seeded in 96‐well plates and incubated for 12 h. The medium was then replaced with fresh medium containing different concentrations of OLNPs or OLNPs@Gel extracts and incubated for 24 h before the Cell Counting Kit‐8 (CCK‐8) assay (Solarbio, China) and live/dead double‐staining assay (Beyotime, China) were conducted. The results of the CCK‐8 assay were determined by measuring the absorbance at 450 nm using a microplate reader. Live/dead staining was visualized by fluorescence microscopy.

### 4.11 Cellular Uptake and Endosomal Escape

To assess the cellular uptake of the circRNA formulation, OCCM‐30 cells were seeded in confocal dishes (2 × 10^4^ cells) overnight. Afterward, OCCM‐30 cells were incubated with PBS, free Cy5‐circRNA, or Cy5‐circRNA‐OLNPs for 6 h. The cells were subsequently washed three times with PBS and imaged with a confocal laser scanning microscope (CLSM) (FV3000; Olympus, Japan) for qualitative analysis and subjected to flow cytometry for quantitative analysis.

To monitor the distribution of circRNAs in cells, OCCM‐30 cells were incubated at 37°C for 1, 3, or 6 h. The nuclei and lysosomes were stained with 4′,6‐diamidino‐2‐phenylindole (DAPI, blue) and LysoTracker Green, respectively, followed by CLSM observation.

### 4.12 ROS, Fe^2^^+^, and Lipid Peroxidation Assays

OCCM‐30 cells were seeded in 12‐well plates at a density of 1 × 10^4^ cells per well and subjected to the indicated treatments for 24 h. Intracellular ROS levels were assessed using a 2′,7′‐dichlorodihydrofluorescein diacetate (DCFH‐DA) probe (Beyotime Biotechnology, China). Fe^2^^+^ levels were measured with RhoNox‐6 (Beyotime Biotechnology, China), and lipid peroxidation was evaluated using BODIPY 581/591 C11 (Beyotime Biotechnology, China).

### 4.13 TEM of Mitochondria

Treated OCCM‐30 cells were collected and fixed with 2.5% glutaraldehyde at 4°C. After being washed with 0.1 M phosphate buffer, the samples were pre‐embedded in 1% agarose and cut into approximately 1‐mm^3^ blocks. The samples were postfixed with 1% osmium tetroxide for 2 h, dehydrated through the use of a graded series of ethanol and acetone, infiltrated, and embedded in epoxy resin. Following polymerization at 60°C for 48 h, ultrathin sections of approximately 70 nm were prepared, mounted on copper grids, and stained with uranyl acetate and lead citrate. The mitochondrial ultrastructure was observed through TEM.

### 4.14 Biochemical Analyses

Cells and tissue samples from each experimental group were lysed, and MDA, GSH, and GSSG levels were measured using assay kits according to the manufacturer's instructions (Solarbio, China). The absorbance was recorded with a microplate reader, and the data were normalized to the total protein concentration.

### 4.15 Immunoprecipitation

Cells were transfected with the indicated plasmids and lysed in immunoprecipitation (IP) lysis buffer containing 50 mm Tris‐HCl (pH 8.0), 150 mm NaCl, 1 mm EDTA, 1% Triton X‐100, and 0.5% sodium deoxycholate supplemented with a protease inhibitor cocktail (Thermo Fisher Scientific, USA) and N‐ethylmaleimide, after which SUMOylation was evaluated. After incubation for 30 min at 4°C, the lysates were clarified through centrifugation, and equal amounts of protein were incubated with the indicated antibodies together with Protein A/G magnetic beads (Thermo Fisher Scientific, USA) overnight at 4°C under gentle rotation. The beads were extensively washed with cold IP lysis buffer, and the immunocomplexes were eluted by boiling in SDS loading buffer. Empty vector‐transfected cells were chosen as blank controls. Immunoprecipitated proteins and corresponding input lysates were subjected to SDS‐PAGE and immunoblotting with the indicated primary antibodies and appropriate secondary antibodies.

### 4.16 Plasmid Transfection

Cells were seeded in culture plates and transfected at 70% confluence with the indicated plasmids, which included Flag‐ALOX15 wild‐type or mutant constructs, HA‐PIAS3, His‐SUMO3, and corresponding empty vectors. Plasmid transfection was achieved using a commercial transfection reagent according to the manufacturer's instructions. In the cotransfection experiments, the total amount of DNA was maintained constant through supplementation with empty vector controls. Cells were then harvested for IP or immunoblotting analysis to evaluate protein interactions, SUMOylation, and protein stability.

### 4.17 SiRNA Transfection

Cells were seeded 12 h before transfection and transfected at approximately 50% confluence. siRNAs targeting mouse *Pias3*, *Rnf4*, and *Ube2g2*, together with a non‐targeting control (si‐NC), were synthesized by GenePharma (Shanghai, China). siRNAs were transfected using siRNA‐Mate plus reagent (G04026, GenePharma) at a ratio of 10 pmol siRNA per 1 µL reagent, with a final siRNA concentration of 30 nm. Cells were maintained in complete high‐glucose DMEM containing 10% FBS and antibiotics. Knockdown efficiency was verified by RT‐qPCR before subsequent experiments. The siRNA sequences are listed in Table S2.

### 4.18 Molecular Docking

The 3D structures of the target proteins were obtained from the Protein Data Bank (PDB) or predicted using AlphaFold when experimental structures were unavailable. Protein–protein docking was conducted using the HDOCK server with default parameters. Docking models with binding energies < −7.0 kcal/mol were retained, and the top‐ranked models were selected based on confidence rankings. Protein–protein interaction interfaces were subsequently visualized and analyzed using PyMOL (version 3.1; Schrödinger, LLC).

### 4.19 SUMOylation, Ubiquitination, and Protein Stability Assays

For SUMOylation assays, OCCM‐30 cells and cementoblast‐enriched primary cells were lysed in lysis buffer supplemented with the deSUMOylation inhibitor N‐ethylmaleimide (NEM; 10 mm; 3876; Sigma–Aldrich, USA). For CHX chase assays, OCCM‐30 cells were treated with CHX (50 µg/mL) and harvested at 0, 4, 8, and 12 h. To determine the degradation pathway of ALOX15, OCCM‐30 cells were treated with the proteasome inhibitor MG132 (0, 5, or 10 µm) or the lysosomal inhibitor chloroquine (CQ; 0, 20, or 50 µm) for 6 h before harvest. For ubiquitination assays, OCCM‐30 cells were treated with MG132 (10 µm) for 6 h before harvest to prevent proteasomal degradation. For pharmacological inhibition of SUMOylation, OCCM‐30 cells were treated with 2‐D08 (50 µm) for 24 h before harvest. Cell lysates were subsequently immunoprecipitated with the indicated antibodies, washed with lysis buffer, and analyzed by immunoblotting.

Candidate SUMOylation sites in ALOX15 were predicted using the GPS‐SUMO (https://sumo.biocuckoo.cn/), SUMOplot (https://www.abcepta.com/sumoplot), JASSA (http://www.jassa.fr/), and DeepSUMO (https://deepsumo.renlab.org/) prediction websites.

### 4.20 Osteoclast Differentiation and OLNPs Carryover Assessment

BMDMs were isolated from mouse femurs and tibias. Bone marrow cells were cultured in alpha minimum essential medium (α‐MEM) supplemented with M‐CSF (50 ng/mL; Novoprotein, China). After 24 h, nonadherent cells were collected and seeded in 24‐well plates at a density of 5 × 10^4^ cells per well. Adherent BMDMs were obtained after 2–3 days of culture.

For osteoclast differentiation, BMDMs were cultured with conditioned media from OCCM‐30 cells subjected to different treatments in the presence of M‐CSF (25 ng/mL) and RANKL (50 ng/mL; Novoprotein, China). The medium was replaced every 24 h. After 5–7 days, the cells were fixed and stained using a TRAP staining kit (Hangzhou Yangming Biotechnology Co., Ltd., China) according to the manufacturer's instructions. TRAP‐positive multinucleated cells containing three or more nuclei were identified as osteoclasts.

To assess potential OLNPs carryover from OCCM‐30‐conditioned medium, BMDMs were additionally exposed to control medium, Cy5‐circRNA‐OLNPs directly, or conditioned medium collected from Cy5‐circRNA‐OLNPs‐treated OCCM‐30 cells, and Cy5 fluorescence was subsequently analyzed by flow cytometry. In a parallel experiment, BMDMs were exposed to control medium, PIAS3‐OLNPs directly, or conditioned medium collected from PIAS3‐OLNPs‐treated OCCM‐30 cells, followed by Western blot analysis of PIAS3 protein expression.

### 4.21 ELISA Analysis of Osteoclastogenic Factors in OCCM‐30 Conditioned Media

Conditioned media were collected from OCCM‐30 cells after the indicated treatments and before supplementation with M‐CSF and RANKL for BMDM differentiation. The samples were centrifuged at 1000 × g for 10 min to remove cellular debris. RANKL, OPG, and M‐CSF levels were measured using commercial ELISA kits (M0644, M0081, M2445; Elabscience, China) according to the manufacturer's instructions. Absorbance was read at 450 nm, and concentrations were calculated from standard curves. Concentrations were normalized to the control group and expressed as fold changes. Three independent biological experiments were performed.

### 4.22 In Vivo Biodistribution of OLNPs

DiR‐labeled OLNPs and OLNPs@Gel were separately injected into the gingival sulcus of the maxillary first molars of the rats. Changes in fluorescence intensity around the teeth were monitored using a small‐animal in vivo optical imaging system (IVIS Spectrum) at 0.5, 6, 24, 48, and 72 h to evaluate the local retention and clearance kinetics of the two formulations in the oral cavity of the rats.

### 4.23 Micro‐CT Analysis

Rat maxillae were fixed in 4% paraformaldehyde at 4°C for 48 h and scanned using a Quantum GX2 micro‐CT system (PerkinElmer, USA) at 50 kV, 88 µA, and a resolution of 36 µm. The scanning field included the maxillary molars and surrounding alveolar bone. 3D reconstruction and morphometric analysis were conducted using Mimics Research 21.0. The root depressions on the pressure side were marked, and their relative volumes were calculated.

### 4.24 Histological Analysis

Following CO_2_ inhalation euthanasia, rat maxillary alveolar bone and periodontal tissues were collected and fixed, decalcified, embedded, and sectioned. H&E staining was implemented to evaluate histopathological changes, TRAP staining was employed to identify osteoclasts, and Prussian blue staining was used to detect iron deposition in periodontal tissues. Immunohistochemistry and immunofluorescence were conducted to evaluate the expression of target proteins. Stained sections were examined under a light microscope and quantified in ImageJ.

### 4.25 Statistical Analysis

Cellular and animal experiments were conducted with random assignment of groups, and outcome evaluations were conducted in a blinded manner. Statistical analyses were conducted in GraphPad Prism 9.5. The data are presented as the mean ± standard error of the mean. Comparisons between two groups were conducted using an unpaired two‐tailed Student's t‐test. Comparisons among multiple groups were conducted using one‐way analysis of variance (ANOVA). Statistical significance was defined as *p* < 0.05.

## Author Contributions

**Yifei Hou**: Writing – review and editing, Writing – original draft, Visualization, Methodology, Investigation, Formal analysis, Conceptualization. **Chongzheng Yan**: Writing – review and editing, Writing – original draft, Visualization, Methodology, Software, Validation. **Xinrui Xu**: Methodology, Data curation, Resources. **Zihan Yang**: Writing – review and editing, Software. **Xinhui Wu**: Writing – original draft, Data curation. **Hongxu Zhao**: Writing – original draft. **Xuan Song**: Supervision, Conceptualization. **Jianing Zhang**: Visualization. **Yijing Li**: Software, Methodology. **Aohua Yang**: Visualization. **Xiamei Lu**: Supervision. **Yi Liu**: Writing – review and editing, Supervision, Project administration, Funding acquisition.

## Conflicts of Interest

The authors declare no conflicts of interest.

## Supporting information




**Supporting File**: advs78455‐sup‐0001‐SuppMat.docx.

## Data Availability

The RNA‐seq data generated in this study have been deposited in the NCBI Gene Expression Omnibus (GEO) under accession number GSE347094. The raw untargeted metabolomics data have been deposited in the OMIX database of the National Genomics Data Center (NGDC), China National Center for Bioinformation (CNCB), under accession number OMIX020579. The key data supporting the findings of this study are included in the manuscript and its Supporting Information. Additional information is available from the corresponding author upon reasonable request.
