# Supplementary material for: PIAS3 CircRNA‐Loaded Lipid Nanoparticles Restore ALOX15 SUMOylation to Suppress Cementoblast Ferroptosis and Attenuate Orthodontically Induced Root Resorption
Source: Adv Sci (Weinh). 2026 Oct 26:e78455. Online ahead of print. doi: 10.1002/advs.78455 (PMC13710712; doi:10.1002/advs.78455)
Supplement: Supplementary file 1 [file ADVS-9999-e78455-s001.docx]

**PIAS3 circRNA-Loaded Lipid Nanoparticles Restore ALOX15 SUMOylation to Suppress Cementoblast Ferroptosis and Attenuate Orthodontically Induced Root Resorption**

Yifei Hou^1,2, †^ | Chongzheng Yan^3, †^ | Xinrui Xu^1,2^ | Zihan Yang^1,2^ | Xinhui Wu^1,2^ | Hongxu Zhao^1,2^ | Xuan Song^1,2^ | Jianing Zhang^1,2^ | Yijing Li^1,2^ | Aohua Yang^1,2^ | Xiamei Lu^1,2^ | Yi Liu^1,2,4, *^

*^1^ Department of Orthodontics, School and Hospital of Stomatology, Cheeloo College of Medicine, Shandong University, Jinan, Shandong 250012, China*

*^2^ Shandong Key Laboratory of Oral Diseases & Shandong Engineering Research Center of Dental Materials and Oral Tissue Regeneration & Shandong Provincial Clinical Research Center for Oral Diseases, Jinan, Shandong 250012, China*

*^3^ School of Pharmacy, Shandong University of Traditional Chinese Medicine, Jinan, Shandong 250355, China*

*^4^ Shenzhen Research Institute of Shandong University, Shenzhen, Guangdong 518055, China*

^*^Correspondence: Yi Liu, Department of Orthodontics, School and Hospital of Stomatology, Cheeloo College of Medicine, Shandong University, No. 44-1 Wenhua Road West, Jinan, Shandong 250012, China. Tel: +86 531-88381630; Fax: +86 531-88382923; Email: [yiliu@sdu.edu.cn.](mailto:yiliu@sdu.edu.cn,)

^†^Yifei Hou and Chongzheng Yan contributed equally to this work.

**Table S1**. RT-qPCR primers.

| **Gene** | **Species** | **Forward Primer** | **Reverse Primer** |
| --- | --- | --- | --- |
| *Gpx4* | Mouse | CCCGATATGCTGAGTGTGGTTTA | TTCTTGATTACTTCCTGGCTCCTG |
| *Slc7a11* | Mouse | CCCTGGCATTTGGACGCTAC | CTCCAGCTGACACTCGTGCTATTTA |
| *Alox15* | Mouse | GGCTCCAACAACGAGGTCTAC | CCCAAGGTATTCTGACACATCCA |
| *Pias3* | Mouse | CCTTGTCCGTGTACCTGGTGAG | GGGTCAGCAGTCAGTTTCTCCTT |
| *Rnf4* | Mouse | TCTTTAGAGCCCGTGGTTGTGG | CTGTCAGCATGGTCTTGGCG |
| *Ube2g2* | Mouse | TCCAGAAGGAATCGTGGCAGG | TGGGAAACTCAGGATGGCGG |
| *Gapdh* | Mouse | TGTGTCCGTCGTGGATCTGA | TTGCTGTTGAAGTCGCAGGAG |
| *Alox15* | Rat | TCGGGACTCGGAAGCAGAAT | CCTTCACAGAAATCCAGTTGCAG |
| *Pias3* | Rat | TCCAGACCATTCCCGAGCAC | TAAAGGGCAGCATCGAAACTCTG |
| *Slc7a11* | Rat | TTTTCTGGCTCCTCACCTCTG | CTGCCGTGCTCACAACTCA |
| *Gapdh* | Rat | TGGTGAAGGTCGGTGTGAAC | TTGCCGTGGGTAGAGTCATA |

**Table S2**. siRNA oligonucleotide sequences.

| **siRNA** | **Species** | **Sense (5′–3′)** | **Antisense (5′–3′)** |
| --- | --- | --- | --- |
| si-*Pias3* | Mouse | GGGACCCUUCUACAAAAACTT | GUUUUUGUAGAAGGGUCCCTT |
| si-*Rnf4* | Mouse | GGAAACUGUUGGAGAUGAATT | UUCAUCUCCAACAGUUUCCTT |
| si-*Ube2g2* | Mouse | CAGUGCAGAGUGUGGAGAATT | UUCUCCACACUCUGCACUGTT |
| si-NC | N/A | UUCUCCGAACGUGUCACGUTT | ACGUGACACGUUCGGAGAATT |

si-NC, non-targeting negative control.

**FIGURE S1** | Fer-1 reduces root resorption and ferroptotic stress without affecting orthodontic tooth movement. (A) Representative sagittal micro-CT images of maxillary molars from the Control, OIRR, and OIRR + Fer-1 groups. (B) Quantification of relative root resorption volume. (C) Quantification of orthodontic tooth movement distance. (D) Immunofluorescence characterization of isolated cementoblast-enriched primary cells by BSP and CD146 staining. Scale bars: 400 μm. (E,F) MDA levels and GSH/GSSG ratios in cementoblast-enriched primary cells, respectively. (G) RhoNox-6 staining showing Fe²⁺ accumulation in cementoblast-enriched primary cells. Scale bars: 200 μm. Data are mean ± standard error of the mean (n = 6); one-way ANOVA; ns, not significant; ***p* < 0.01; ****p* < 0.001; *****p* < 0.0001.

**FIGURE S2** | Workflow for isolation and culture of cementoblast-enriched primary cells.

**FIGURE S3** | Fer-1 attenuates compression-induced ferroptosis in OCCM-30 cells. (A) Western blot analysis of GPX4 expression in OCCM-30 cells subjected to compressive loading for 0, 6, 12, 24, and 48 h. (B) Representative bright-field images of OCCM-30 cells under control and compression conditions. Scale bars: 500 μm. (C) Western blot analysis of ACSL4, SLC7A11, and FTH1 in Control, Compression + PBS, and Compression + Fer-1-treated OCCM-30 cells. (D) Flow cytometric analysis of BODIPY 581/591 C11 fluorescence. (E,F) MDA levels and GSH/GSSG ratios in OCCM-30 cells, respectively. (G) BODIPY 581/591 C11 staining showing lipid oxidation and reduction states. Scale bars: 200 μm. (H) RhoNox-6 staining showing Fe²⁺ accumulation. Scale bars: 200 μm. G1, Control; G2, Compression + PBS; G3, Compression + Fer-1. Data are mean ± standard error of the mean (n = 6); one-way ANOVA; **p* < 0.05, ***p* < 0.01, ****p* < 0.001.

**FIGURE S4** | GO enrichment analysis of DEGs in OIRR periodontal tissues.

**FIGURE S5** | Validation of PIAS3–ALOX15 dysregulation in cementoblast-enriched primary cells from OIRR rats. RT-qPCR analysis of (A) *Alox15*, (B) *Pias3*, and (C) *Slc7a11* mRNA expression in cementoblast-enriched primary cells isolated from Control and OIRR rats. (D) Western blot analysis of ALOX15, PIAS3, and SLC7A11 protein expression in cementoblast-enriched primary cells. Data are mean ± standard error of the mean (n = 6); unpaired two-tailed Student’s t-test; *****p* < 0.0001.

**FIGURE S6** | Time-dependent ALOX15 protein expression in compressed OCCM-30 cells. (A) Representative Western blot bands of ALOX15 in OCCM-30 cells subjected to compressive force for 0, 6, 12, 24, and 48 h. (B) Densitometric quantification of relative ALOX15 protein abundance. Data are mean ± standard error of the mean (n = 3); one-way ANOVA; ns, not significant; ****p* < 0.001, *****p* < 0.0001.

**FIGURE S7** | Untargeted metabolomics reveals ferroptosis-related metabolic remodeling in OIRR. (A) Volcano plot of differential metabolites. (B) MSEA of arachidonic acid and glutathione metabolism. (C) Heatmap of selected ferroptosis-associated metabolites. (D) ALOX15-centered correlation network of differential metabolites.

**FIGURE S8** | Representative immunoblots of co-IP assays in cells co-transfected with HA-PIAS3 and Flag-ALOX15.

**FIGURE S9** | PIAS3 inversely regulates ALOX15 protein stability. (A, B) CHX chase analysis and quantification of ALOX15 after PIAS3 overexpression. (C) RT-qPCR analysis of *Pias3* mRNA expression in OCCM-30 cells transfected with si-PIAS3 or non-targeting negative-control siRNA (si-NC). (D, E) CHX chase analysis and quantification of ALOX15 after PIAS3 knockdown. Data in (C) are presented as mean ± standard error of the mean (n = 3); unpaired two-tailed Student’s t-test; ***p* < 0.01.

**FIGURE S10** | PIAS3 knockdown enhances compression-induced ALOX15 accumulation without increasing *Alox15* transcription. (A) RT-qPCR analysis of *Alox15* mRNA expression in OCCM-30 cells transfected with si-NC or si-PIAS3. (B) Western blot analysis of endogenous ALOX15 and PIAS3 in OCCM-30 cells transfected with si-NC or si-PIAS3, with or without compressive loading. (C) Western blot analysis of Flag-ALOX15 and PIAS3 in Flag-ALOX15–expressing OCCM-30 cells transfected with si-NC or si-PIAS3, with or without compressive loading. Data are mean ± standard error of the mean (n = 3); unpaired two-tailed Student’s t-test; ns, not significant.

**FIGURE S11** | Molecular docking of PIAS3 and ALOX15. PIAS3 is shown as the receptor (green) and ALOX15 as the ligand (pink). Dashed lines indicate predicted noncovalent interactions.

**FIGURE S12** | Western blot analysis of SENP1, SENP2, SENP3, and SENP5 in OCCM-30 cells.

**FIGURE S13** | Prediction and evolutionary conservation of ALOX15 SUMOylation sites. (A) Candidate SUMOylation sites predicted using JASSA, SUMOplot, DeepSUMO, and GPS-SUMO. (B) Multiple-sequence alignment of the N-terminal ALOX15 region across 10 mammalian species; the conserved Lys50 residue is highlighted in red.

**FIGURE S14** | RNF4 mediates PIAS3-induced K48-linked ubiquitination of ALOX15. (A) Endogenous co-immunoprecipitation analysis of the interaction between ALOX15 and RNF4 in OCCM-30 cells. ALOX15 immunoprecipitates and IgG controls were immunoblotted for ALOX15 and RNF4. (B) Endogenous co-immunoprecipitation analysis of the interaction between ALOX15 and UBE2G2 in OCCM-30 cells. ALOX15 immunoprecipitates and IgG controls were immunoblotted for ALOX15 and UBE2G2. (C,D) RT-qPCR analysis of *Rnf4* and *Ube2g2* mRNA expression, respectively, in OCCM-30 cells transfected with the corresponding targeting siRNA or non-targeting negative-control siRNA (si-NC). (E) Immunoprecipitation analysis of K48-linked ubiquitination of ALOX15 after PIAS3 overexpression and siRNA knockdown of RNF4 or UBE2G2 in MG132-treated OCCM-30 cells. Data in (C,D) are presented as mean ± standard error of the mean (n = 3); unpaired two-tailed Student’s t-test; **p* < 0.05, ***p* < 0.01.

**FIGURE S15** | Molecular docking analysis of the rat ALOX15–RNF4 interaction. Predicted docking model of rat ALOX15 and RNF4, with ALOX15 shown in pink and RNF4 shown in green. Enlarged views highlight representative interaction interfaces and predicted hydrogen bonds between the two proteins, with distances indicated in Å.

**FIGURE S16** | Molecular docking analysis of the human ALOX15–RNF4 interaction. Predicted docking model of human ALOX15 and RNF4, with ALOX15 shown in pink and RNF4 shown in green. Enlarged views show representative interaction interfaces and predicted hydrogen bonds between the two proteins, with distances indicated in Å.

**FIGURE S17** | Synthesis and structural characterization of OA-C12. (A) Synthetic route for the uridine-derived ionizable lipid OA-C12. (B) ¹H NMR spectrum of the final product.

**FIGURE S18** | Optimization of OLNPs composition for circRNA delivery. (A) Molar compositions of the 16 candidate formulations. (B) Proportion of EGFP-positive OCCM-30 cells after EGFP circRNA delivery. Data are mean ± standard error of the mean (n = 3).

**FIGURE S19** | Release and degradation characteristics of the OLNPs@Gel system. (A) Cumulative release of PIAS3 circRNA from PIAS3-OLNPs@Gel at pH 6.5 and 7.4. (B) Degradation profiles of Gel and OLNPs@Gel in artificial saliva.

**FIGURE S20** | Cellular uptake and cytocompatibility of circRNA-OLNPs. (A) Flow cytometry histograms and (B) MFI of Cy5-circRNA uptake. G1, PBS; G2, free Cy5-circRNA; G3, Cy5-circRNA-OLNPs. (C) Viability of OCCM-30 cells treated with different concentrations of circRNA-OLNPs. Data are mean ± standard error of the mean (n = 3); one-way ANOVA; ns, not significant; *****p* < 0.0001.

**FIGURE S21** | In vitro cytocompatibility of the OLNP-based delivery systems. Representative live/dead staining of OCCM-30 cells treated with blank OLNPs, PIAS3-OLNPs, or PIAS3-OLNPs@Gel. Scale bar: 500 μm.

**FIGURE S22** | PIAS3-OLNPs restore ferroptosis-related protein and mRNA expression in compressed OCCM-30 cells. (A–C) Quantification of GPX4, SLC7A11, and ALOX15 by Western blot analysis. (D–F) RT-qPCR analysis of *Gpx4*, *Slc7a11*, and *Alox15*. G1, Control; G2, Compression + PBS; G3, Compression + free circRNA; G4, Compression + blank OLNPs; G5, Compression + PIAS3-OLNPs. Data are mean ± standard error of the mean (n = 3); one-way ANOVA; **p* < 0.05, ***p* < 0.01, ****p* < 0.001, *****p* < 0.0001.

**FIGURE S23** | PIAS3-OLNPs attenuate the osteoclastogenic activity of conditioned medium from compressed cementoblasts. (A–C) Fold changes in RANKL, OPG, and M-CSF concentrations in OCCM-30-derived conditioned medium. (D) RhoNox-6 staining showing Fe²⁺ accumulation in BMDMs exposed to conditioned medium from different OCCM-30 treatment groups. Scale bars: 200 μm. G1, Control; G2, Compression + PBS; G3, Compression + free circRNA; G4, Compression + blank OLNPs; G5, Compression + PIAS3-OLNPs; G6, Compression + Fer-1. Data are mean ± standard error of the mean (n = 3); one-way ANOVA; **p* < 0.05, ***p* < 0.01, ****p* < 0.001, *****p* < 0.0001.

**FIGURE S24** | Assessment of potential PIAS3-OLNPs carryover to BMDMs. (A) Representative flow cytometry histograms of Cy5 fluorescence in BMDMs exposed to control medium (Control), Cy5-circRNA-OLNPs directly (Direct), or conditioned medium collected from Cy5-circRNA-OLNPs-treated OCCM-30 cells (CM). (B,C) Western blot analysis and quantification of PIAS3 expression in BMDMs. G1, Control; G2, direct PIAS3-OLNPs exposure; G3, conditioned medium from PIAS3-OLNPs-treated OCCM-30 cells. Data are mean ± standard error of the mean (n = 3); one-way ANOVA; ns, not significant; *****p* < 0.0001.

**FIGURE S25** | Systemic biocompatibility and local retention of the OLNPs@Gel platform. (A) Representative H&E-stained images of heart, liver, spleen, lung, and kidney after local treatment. Scale bar: 100 μm. (B) Representative serial in vivo fluorescence images acquired at 0.5, 6, 24, 48, and 72 h after gingival-sulcus administration of DiR-labeled OLNPs or OLNPs@Gel.

**FIGURE S26** | (A) Representative sagittal micro-CT images of maxillary molars from different treatment groups. (B) Quantification of orthodontic tooth movement distance. G1, Control; G2, OIRR + PBS; G3, OIRR + blank OLNPs@Gel; G4, OIRR + PIAS3-OLNPs; G5, OIRR + PIAS3-OLNPs@Gel. Data are mean ± standard error of the mean (n = 6); one-way ANOVA; ns, not significant; *****p* < 0.0001.

**FIGURE S27** | PIAS3-OLNPs@Gel suppress ferroptosis in cementoblast-enriched primary cells isolated from OIRR rats. (A) Western blot analysis of ALOX15 and PIAS3 in cementoblast-enriched primary cells. (B) Western blot analysis of ACSL4 and GPX4 in cementoblast-enriched primary cells. (C,D) MDA levels and GSH/GSSG ratios in cementoblast-enriched primary cells, respectively. (E) BODIPY 581/591 C11 staining showing lipid oxidation and reduction states in cementoblast-enriched primary cells. Scale bars: 200 μm. (F) RhoNox-6 staining showing Fe²⁺ accumulation in cementoblast-enriched primary cells. Scale bars: 200 μm. G1, Control; G2, OIRR + PBS; G3, OIRR + PIAS3-OLNPs@Gel. Data are mean ± standard error of the mean (n = 6); one-way ANOVA; **p* < 0.05, ***p* < 0.01.

**FIGURE S28** | PIAS3-OLNPs@Gel promote ALOX15 SUMOylation and K48-linked ubiquitination in cementoblast-enriched primary cells. (A) Endogenous ALOX15 immunoprecipitation followed by immunoblotting for SUMO2/3 in cementoblast-enriched primary cells. Input lysates were immunoblotted for ALOX15 and PIAS3. (B) Endogenous ALOX15 immunoprecipitation followed by immunoblotting for K48-linked ubiquitin in cementoblast-enriched primary cells. Input lysates were immunoblotted for ALOX15 and PIAS3. G1, Control; G2, OIRR + PBS; G3, OIRR + PIAS3-OLNPs@Gel.
